# Environmental, individual and social traits of free-ranging raccoons influence performance in cognitive testing

**DOI:** 10.1242/jeb.243726

**Published:** 2022-09-22

**Authors:** Lauren A. Stanton, Eli S. Bridge, Joost Huizinga, Sarah Benson-Amram

**Affiliations:** ^1^ Department of Zoology and Physiology, University of Wyoming, Laramie, WY 82071, USA; ^2^ Program in Ecology, University of Wyoming, Laramie, WY 82071, USA; ^3^ Oklahoma Biological Survey, University of Oklahoma, Norman, OK 73019, USA; ^4^ OpenAI, San Francisco, CA 94110, USA

**Keywords:** Carnivore, Cognition, Flexibility, Learning, RFID, Urban

## Abstract

Cognitive abilities, such as learning and flexibility, are hypothesized to aid behavioral adaptation to urbanization. Although growing evidence suggests that cognition may indeed facilitate persistence in urban environments, we currently lack knowledge of the cognitive abilities of many urban taxa. Recent methodological advances, including radio frequency identification (RFID), have extended automated cognitive testing into the field but have yet to be applied to a diversity of taxa. Here, we used an RFID-enabled operant conditioning device to assess the habituation, learning and cognitive flexibility of a wild population of raccoons (*Procyon lotor*). We examined how several biological and behavioral traits influenced participation and performance in testing. We then compared the cognitive performance of wild raccoons tested in natural conditions with that of wild-caught raccoons tested in captivity from a previous study. In natural conditions, juvenile raccoons were more likely to habituate to the testing device, but performed worse in serial reversal learning, compared with adults. We also found that docile raccoons were more likely to learn how to operate the device in natural conditions, which suggests a relationship between emotional reactivity and cognitive ability in raccoons. Although raccoons in both captive and natural conditions demonstrated rapid associative learning and flexibility, raccoons in captive conditions generally performed better, likely owing to the heightened vigilance and social interference experienced by raccoons in natural conditions. Our results have important implications for future research on urban carnivores and cognition in field settings, as well as our understanding of behavioral adaptation to urbanization and coexistence with urban wildlife.

## Teaser

**Highlighted Article:** Raccoons are a highly adaptive species anecdotally known for their intelligence. Advanced technologies used to test wild raccoon cognition found new insights into their behavior and cognitive abilities.

## INTRODUCTION

Cognition, the process by which organisms acquire, process, store and act on information from the environment, is central to an organism's ability to overcome social and ecological challenges (Shettleworth, 2010). Cognition is hypothesized to be especially important for organisms living in novel, complex and moderately predictable environments, where they must quickly process and respond appropriately to various environmental stimuli and conditions (Mettke-Hofmann, 2014). For these reasons, cognition has been proposed as an important mechanism for the persistence of wildlife in urban environments (Sol et al., 2013). Cities are characterized by tremendous heterogeneity across spatial and temporal scales, presenting organisms with novel and diverse stimuli and conditions (Griffin et al., 2017; Lambert et al., 2021). Animals that can use cognition to capitalize on anthropogenic resources while evading threats are more likely to survive in urban environments. Yet the degree to which cognition plays a role in urban persistence remains largely undetermined and is likely related to several factors, such as a particular species' ecology, stage of urban invasion and perception/tolerance by humans (Barrett et al., 2019; Sayol et al., 2020; Wright et al., 2010). As such, we are very much still in the early stages of understanding the complex link between animal cognition and urban living (Lee and Thornton, 2021).

Several cognitive abilities have been proposed as particularly important for urban wildlife. Habituation, for example, is a non-associative form of learning that allows animals to decrease their responsiveness to harmless stimuli with increased exposure (Blumstein, 2016). Although habituation to humans may be an adaptive strategy for some urban species that have frequent, innocuous interactions with humans, it can also be problematic for species perceived as a nuisance by humans (Barrett et al., 2019; Schell et al., 2021). Another example is associative learning, which allows animals to form an association between cues, or between a particular stimulus and a response (Papini, 2002). This type of learning is highly conserved across different species, and can be adaptive when aiding animals in fitness-related tasks, such as finding food and mates or avoiding predation (Ginsburg and Jablonka, 2010). However, associative learning speed and ability have been shown to vary among individuals and species, and the causes and consequences of such variation are not fully understood (Morand-Ferron, 2017; Sih and Del Giudice, 2012). Finally, behavioral flexibility is a broad term used to describe an animal's ability to change its behavior in response to change and variation in its environment (Audet and Lefebvre, 2017; Lea et al., 2020). It is often cited as one of the most critical mechanisms for urban invasion and persistence, as it allows animals to respond appropriately to new and diverse obstacles and opportunities in a flexible manner (Sol et al., 2013). Despite our growing sense of the importance of cognitive abilities such as habituation, learning and behavioral flexibility for animals living in urban environments, direct measures of such abilities remain limited.

Our current understanding of the role of cognition in urban living is largely based on indirect metrics of cognition (e.g. relative brain size) and comparisons of cognition between urban and non-urban populations following predictions outlined by the cognitive buffer hypothesis (CBH). The CBH states that large brains facilitate domain-general cognitive abilities that allow animals to modulate new and flexible behaviors that enhance their survival and fitness (Allman et al., 1993; Sol, 2009). In line with this hypothesis, several studies have found correlational evidence linking large relative brain size to invasion success across several taxa (amphibians and reptiles: Amiel et al., 2011; birds: Sol et al., 2005; mammals: Sol et al., 2008), and, in some cases, the invasion of urban environments by birds (Carrete and Tella, 2011; Sayol et al., 2020). There is also some experimental evidence demonstrating that urban individuals have superior learning and problem-solving abilities compared with their non-urban counterparts (e.g. birds: Audet et al., 2016). However, results from these studies are not always in agreement and are generally limited to birds (Griffin et al., 2017; Lee and Thornton, 2021; but see Johnson-Ulrich et al., 2021; Chow et al. 2021). Given that the use of urban spaces, resources and stimuli differs greatly across species, this taxonomic bias towards birds currently limits our understanding of the relationship between cognition and urban living (Lee and Thornton, 2021).

In addition to current taxonomic limitations, the causes and consequences of individual variation in cognitive ability represents a significant gap in our knowledge (Boogert et al., 2018). Moreover, there are many individual-level traits that influence participation and performance in cognitive testing, which can complicate interpretation of results (Thornton and Lukas, 2012). For instance, studying animal cognition in the field is a challenging yet valuable approach, as animals living in natural conditions are exposed to environmental pressures and socio-ecological challenges that are difficult to replicate in captive and laboratory conditions for many species (Griebling et al., 2022). Participation of free-ranging animals is voluntary, however, and therein subject to various motivational factors, including energetic demands and hunger (Morand-Ferron et al., 2015a). In addition, certain behavioral traits, such as docility and boldness, may also increase the likelihood of participation, or even determine performance, in cognitive tasks (Boogert et al., 2018; Hare et al., 2002; Sih and Del Giudice, 2012). Because individual variation is not always considered or accounted for, especially when data collected from individuals are pooled to facilitate interpopulation comparisons such as urban versus non-urban, within-population variation in cognitive ability related to behavioral and biological traits such age, sex or personality in urban wildlife is not well known (Lee and Thornton, 2021). Thus, directly assessing cognitive abilities of importance in a broader diversity of urban species while identifying sources of individual variation can greatly improve our understanding of how animals persist in urban environments.

Raccoons (*Procyon lotor*) are medium-sized, generalist carnivores that demonstrate incredible success in novel and urban environments yet have been understudied within the fields of animal behavior and cognition. The raccoon's native range in North America is currently expanding, and raccoons are now in many parts of Europe and Asia owing to human-mediated introductions (Hadidian et al., 2010; Timm et al., 2008), trends that are only expected to increase globally as increasing temperatures associated with climate change create more favorable conditions for highly adaptive species such as raccoons (Louppe et al., 2019). Historical records of raccoon behavior and distribution suggest that raccoon exploitation of urban areas has been longstanding, and raccoons are commonly found in high densities in North American cities today (Hadidian et al., 2010; Zeveloff, 2002). Although the cognition of raccoons has been generally underexplored, raccoons are well known for their intelligence and innovative foraging strategies. This perception not only stems from contemporary observations of raccoon behavior, but is also reflected in notes by early naturalists (e.g. Audubon and Bachman, 1851) as well as in some North American Indigenous cosmologies (Justice, 2021; Pacini-Ketchabaw and Nxumalo, 2015). In addition, recent studies have provided new insights into raccoon brain morphology (Jacob et al., 2021; Jardim-Messeder et al., 2017) and demonstrated the rapid learning, flexibility and innovative problem-solving abilities of raccoons (Daniels et al., 2019; Stanton et al., 2017, 2021), all of which point to enhanced cognitive ability. Finally, because of their proficiency at exploiting anthropogenic resources, as well as their status as a zoonotic disease vector, raccoons are commonly involved in conflict with humans and confronted by a variety of lethal and non-lethal management strategies not experienced by some other urban species (Gehrt, 2004; Hadidian et al., 2010). Thus, investigation of raccoon cognition and behavior has the potential to uncover greater insights into the traits that facilitate persistence in novel and urban environments.

Here, we investigated the behavior and cognitive flexibility of wild, free-ranging raccoons in an urban environment using an automated testing device adapted for field use. Although there are many ways to study behavioral flexibility, flexibility in cognition is traditionally assessed using a test known as reversal learning (Mackintosh et al., 1968). In the reversal-learning paradigm, a previously learned reward association is reversed, and the ability of the animal to correctly respond to the new reward contingency by changing its behavior remains a widely accepted measure of cognitive, and thereby behavioral, flexibility (Audet and Lefebvre, 2017; Izquierdo et al., 2017). In this study, we used a spatial reversal-learning task, in which raccoons were required to discriminate between right versus left stimuli to receive an automated food reward. We assessed cognitive flexibility by quantifying the number of errors raccoons made during their reversals and identified several potential predictors of performance. Following increasingly common research methods employed in studies of free-ranging birds (e.g. Aplin et al., 2015; Bridge et al., 2019; Cauchoix et al., 2017), we used radio frequency identification (RFID) to identify individual raccoons within our study population and to collect repeated measures of each individual's performance across a series of multiple, successive reversal events (i.e. serial reversal learning). In addition to assessing cognitive flexibility, our automated testing device represents a novel, anthropogenically derived source of food. Thus, we also consider our study to be an analogue that demonstrates how wild raccoons living in an urban landscape respond to novel but potentially risky opportunities in their environment.

Our aims were to evaluate how individual traits and testing conditions affected the behavior and cognitive performance of wild raccoons within our urban study population. We did this in two ways. First, we tested how individual biological and behavioral traits predicted habituation to the automated testing device, learning to operate the device and performance in reversal learning. Although our study was generally exploratory owing to its novelty, we did expect to find individual variation in the response of raccoons to testing based on the perceived risks and rewards associated with the testing device. Specifically, we predicted increased habituation and use of the device by raccoons that were in poorer body condition (i.e. in greater nutritional deficit), younger (i.e. more naïve), bolder (i.e. more aggressive and risk prone) and more social (i.e. more likely to be influenced by the presence of conspecifics). We also expected to find differences in the reversal-learning performance of juveniles and adults potentially stemming from differences in developmental effects (e.g. juvenile cognitive flexibility is underdeveloped in comparison with adults) or simply their general interest in the task (e.g. juveniles tend to show increased interest and thereby success) (Kayser et al., 2021). Second, we compared the performance of wild raccoons tested in natural conditions with the performance of wild raccoons previously tested in captive conditions (Stanton et al., 2021). We expected that raccoons tested in natural conditions would have to contend with interruptions and competing demands on their time and attention not experienced by raccoons tested in captive conditions (e.g. vigilance, social interactions) (Benson-Amram et al., 2013), and therefore predicted that wild raccoons tested in natural conditions would perform worse in comparison with wild raccoons tested in captivity.

## MATERIALS AND METHODS

### Study subjects and site

This research was conducted with wild raccoons [*Procyon lotor* (Linnaeus 1758)] living in the city of Laramie, Wyoming, USA. Located in southeastern Wyoming, Laramie sits on a high elevation prairie (2200 m) between the Laramie Range and the Medicine Bow Mountains. It encompasses approximately 29 km^2^, is home to approximately 32,000 people, and generally experiences long, cold winters and short, mild summers (US Census Bureau, 2021; Western Regional Climate Center, 2016). We characterize this population of raccoons as urban based on the density of humans living in Laramie (approximately 1711 per square mile; US Census Bureau, 2021) as well as the presence of built structural components (e.g. houses, buildings, roads) and other anthropogenic entities (e.g. garbage, pets), albeit at a smaller scale than other major US cities (Moll et al., 2019).

To identify free-ranging raccoons, we conducted annual trapping at locations within Laramie where we had confirmed raccoon presence via trail camera footage. Trapping took place from August 2015 until September 2019 during warmer months when the overnight lows were above 32°F (typically during August and September, but trapping was opportunistic and varied annually from May to October). Tomahawk live traps (81.28×25.4×25.4 cm, length×width×height) baited with wet cat food were set at dusk and checked between the hours of 03:00 and 05:00 h the following morning. We transported captured raccoons in the Tomahawk traps from their capture location to the University of Wyoming's Red Buttes Environmental Laboratory for processing, located 13 km south of Laramie and approximately 15 min from any of our trapping sites. We continued trapping at each site until we captured either no raccoons or only trapped recaptured individuals for multiple (3–7) nights at that location during a trapping year.

At the Red Buttes Environmental Laboratory, we immobilized raccoons in the trap with an intramuscular injection of Telazol^®^ (100 mg ml^−1^) using a 9–11 mg kg^−1^ dose, depending on the size of the individual. Once a raccoon was successfully immobilized, we removed the raccoon from its trap for processing. We determined the sex and approximate age of each raccoon by inspecting their teeth, genitals, reproductive status and body size. Specifically, individuals were classified as an adult if their teeth showed signs of wear, they weighed at least 5 kg and they showed signs of having bred previously and/or during the current season (e.g. teats large and dark in color, testicles descended, fur missing from testicles) (Grau et al., 1970; Pitt et al., 2008). We also weighed each raccoon and measured its body length from the tip of its nose to the base of its tail. We then performed a linear regression of these two measures to calculate a body condition score (BCS) for each individual (i.e. residuals from a regression of body mass and body length) (Schulte-Hostedde et al., 2005). We injected every raccoon with a subcutaneous passive integrated transponder (PIT) tag between the raccoon's shoulder blades, which allowed us to identify every individual via RFID methods. Once the raccoons were fully recovered from the effects of Telazol^®^, we offered each raccoon food and water before transport back to its capture site for release.

### Behavioral assessments

To assess individual variation in traits that might influence participation or performance in cognitive testing, we performed standardized assessment of raccoon behavior at several points during capture and processing in the 2016–2019 trapping years. These assessments were adapted from similar studies of mammals that measured docility as a reaction to humans during trapping and handling, which is generally considered to be related to other personality traits such as aggression and boldness (Petelle et al., 2013; Réale et al., 2000). The production of aggressive vocalizations (emittance of growls, snarls, snorts, hisses and barks; Sieber, 1984), movement (the raccoon shifting its body in the trap) and contact with humans (the raccoon sticks its arm out of the trap and makes contact with trap handler) were recorded during human–raccoon encounters at four distinct phases: capture (when raccoons were collected from the field and transported to Red Buttes), immobilization (when raccoons were approached and injected with Telazol^®^), feeding (when raccoons were presented with food and water after recovery from immobilization) and release (when raccoons were transported back to their capture location and released). An observer recorded the production of these three behaviors at multiple standardized points during each of the four phases (Table S1), and the humans interacting with the raccoons at each phase (i.e. trap handlers) were instructed to confirm the production of each behavior. Thus, all behaviors were agreed upon by handlers and observers. The frequency of behaviors was aggregated into three categories: behavior never produced (0), behavior produced in less than half of the observations made (1), or behavior produced in more than half of the observations made (2). If an individual was recaptured multiple times in a single year, it was released, and therefore each individual was only assessed once during each trapping year. Given that raccoon personality has yet to be empirically assessed, we based our predictions on what is known about docility in other mammalian species (e.g. Petelle et al., 2013; Réale et al., 2000), and therefore defined less docile raccoons as individuals that vocalized, moved and made physical contact with humans more frequently than more docile individuals.

### Trappability

The readiness of an animal to be trapped is also often associated with boldness (Carter et al., 2012; Garamszegi et al., 2009; Réale et al., 2000). Therefore, we also calculated a trappability score for each individual captured during 2016–2019 by counting the number of times an individual was captured at a single site within a trapping year and dividing it by the total number of nights that traps were open at that site during a trapping year (Réale et al., 2000). If an individual was captured at more than one site during a trapping year (uncommon, *N*=11 raccoons over five trapping years), then its trapping score was averaged between the different sites. Raccoons that were not trapped during a trapping year but were confirmed alive and present at a trapping site (via PIT tag detection after trapping efforts finished) were assigned a score of 0 for years when they were not trapped. It is possible that some animals were not captured on certain nights because all traps were occupied by other individuals; however, such trapping saturation was uncommon and occurred only on a minority of nights from 2016 to 2019 (9% of trapping nights across years). In addition, we typically set several traps at a site for over 3 weeks, providing ample opportunity for capture.

### RFID-enabled operant conditioning device

We tested raccoons using an automated operant conditioning device similar to a classic ‘Skinner box’ (Skinner, 1938). The device was made of plywood (84.5×57.8×80.0 cm, length×width×height) and contained a large copper antenna that was placed at an ∼45 deg angle within the walls of the device. The antenna was attached to a long-range RFID reader (Biomark^®^ IS1001 Reader), which detected the presence of PIT tags within its reading range (i.e. tags within the device or approximately 30 cm outside of the device) and was used to deliver tests specific to an individual while keeping a record of that individual's progress across time. The device had two LED buttons that could be pressed to indicate a selection. Pushing on the correct button (positive stimulus) would automatically trigger the release of a small amount of dry dog kibble (reward) from a food-dispensing chute located midway between the buttons. In contrast, pushing the incorrect button (negative stimulus) would emit a low-pitched sound accompanied by a brief (2 s) timeout in which the LED lights shut off and the device became unresponsive to additional button pushes (Movie 1). We restricted the timeout period to 2 s so that we could avoid abandonment of a trial, which was previously observed in captivity when longer timeout periods were used (Stanton et al., 2021). The device was powered by a 12 V sealed, rechargeable battery and we used a Raspberry Pi computer board to control the RFID antenna, LED button interface, motor control and data-logging requirements. We placed one or two devices at each study site depending on the amount of space available and the anticipated volume of raccoons at each site, which we based on trapping success specific to that site. When two devices were used simultaneously, raccoons were assigned to only one of the boxes, such that only one box would respond to the raccoon's PIT tag. During initial testing of the boxes, we experienced minor malfunctions and had to remove the devices from the field for repair. Also, raccoons would occasionally remove the buttons from the testing interface, which we were able to repair in the field the morning following a testing session. Data collected during times of malfunction, button destruction or heavy social interference were not included in the dataset (*N*=9 trials).

### Reversal-learning protocol

Trials were conducted during October–November 2018 and May–October 2019 at four study sites spaced 1–3.5 km apart (mean=1.84 km). All four study sites, which also served as trapping sites, were located on the west side of Laramie close to a greenbelt and the Laramie River (Supplementary Materials and Methods). Raccoons were presented with a two-choice spatial paradigm similar to the methods of Cauchoix et al. (2017) and Stanton et al. (2021). Our testing protocol was fully automated and therefore did not require active experimenter involvement. The devices were switched on at dusk and switched off the following morning, so that testing occurred at night, when raccoons are typically most active. To motivate participation and habituate the raccoons to the lights and sounds of the device, raccoons would receive up to three rewards for simply approaching the box every night (Movie 2). This provided raccoons with an opportunity to learn that the devices were a source of food and did not pose a risk. We placed inaccessible food within the LED buttons to encourage the raccoons to explore the buttons and shape the button-pushing behavior (Morand-Ferron et al., 2015b). During the first few nights that the boxes were in the field, we also placed a small amount of high-value food rewards (e.g. sardines, peanut butter) within and around the device to attract raccoons to the device and again aid in shaping the button-pushing behavior.

Each raccoon began with a training phase in which they were rewarded for pushing on either button (e.g. on the left or right side) 11 times. This allowed raccoons to begin to form an association between pushing the buttons and receiving a food reward. Whichever side the raccoon pushed more frequently during this training phase was considered to be its preferred side and is why we chose an odd number of training pushes (i.e. 11 pushes). Once a raccoon completed its training phase by pushing on the buttons 11 times, it was automatically advanced into trials where it was rewarded only for correct selections and received a brief timeout for incorrect selections. The initial correct response was determined by the raccoon's preferred side so that raccoons were reinforced for pushing the button they selected more frequently during their training phase. We employed a 90% learning criterion in which raccoons were required to make nine correct selections out of 10 consecutive selections before a reversal was initiated. When the raccoon met the 90% criterion, the device automatically reversed the reward contingency so that the opposite button (i.e. right or left side) became the new correct selection. The number of incorrect selections (i.e. number of errors) was counted for each reversal event. To ensure that raccoons in the area had an opportunity to encounter the boxes and complete serial reversals, we conducted trials for 12–20 nights (mean=17) at a study site before moving the devices to a new study site in the interest of time and increasing our sample size. We found this to be a sufficient amount of testing time at a site because it was uncommon for new individuals to visit a site after 10 days of testing and most active participants could complete multiple serial reversals during this time. Raccoons were not limited in the minimum or maximum number of reversals that they could complete during this time, and our goal was to obtain as many reversals as possible for every individual.

### Captive trials

We recently conducted a similar reversal-learning study with wild-caught raccoons at the USDA National Wildlife Research Center (NWRC) in Fort Collins, Colorado, USA (Stanton et al., 2021). Raccoons (*N*=11 adults) were trapped in the Laporte, CO, area as a part of ongoing USDA research and had been in captivity for 16–21 months prior to the start of cognitive testing. Thus, we were interested in comparing the results of these wild-caught raccoons tested under captive, controlled conditions (i.e. ‘captive raccoons’) with those of the wild, free-ranging raccoons tested under natural conditions in Laramie. Captive raccoons were presented with the same two-choice spatial paradigm as in the present study and they were required to meet a 90% learning criterion. There are a few differences between protocols inherent to each testing condition, which include presence/absence of conspecifics, the ages of participants, and the timing and delivery of trials (see Supplementary Materials and Methods). Additional details regarding the captive study can be found in Stanton et al. (2021).

### Statistical analyses

All analyses were performed using R (https://www.r-project.org/). *Post hoc* comparative analyses of factors in generalized linear models (GLMs) and generalized linear mixed models (GLMMs) were performed using contrast tests with the package emmeans (https://CRAN.R-project.org/package=emmeans). Model selection was performed using Akaike's information criterion corrected for small sample sized (AICc) with the package MuMIn (https://CRAN.R-project.org/package=MuMIn). Data were transformed to achieve normality where necessary and model summary tables are provided in the supplementary material (Tables S1–S5). Unlike testing conducted in laboratory and captive settings, our sample size, and thus power for analyses, was based on opportunistic visitation and participation by free-ranging individuals.

#### Biological and behavioral traits

We assessed whether age and sex (independent variables) were related to BCS, trappability and docility (dependent variables) using GLMs. We also wanted to know how these traits may have influenced participation in our study. However, raccoons were not always trapped the same year they were tested, and we therefore only included biological variables that were repeatable across trapping years. If a raccoon was not captured during the year it was tested (*N*=16), we assigned an ‘age at testing’ based on our knowledge of its most recent age: if a raccoon had been trapped as an adult, it was always considered an adult thereafter. If the raccoon was trapped as a juvenile and 2 years had passed since it was trapped, we re-classified that individual as an adult at the time of testing; if not, it was still considered a juvenile.

Docility was first measured by calculating the repeatability of behaviors (i.e. vocalization, movement and contact) within a single trapping event (i.e. across each of the four testing phases: capture, immobilization, feeding and release) and, when applicable, across trapping events (i.e. recaptures across trapping years). When calculating the repeatability of behaviors across trapping events, behavioral scores were aggregated across the four trapping phases so that an individual had a single score for each behavior for a trapping year. Thus, within a testing phase, an individual could score either a 0, 1 or 2 for each behavior in each phase, and when aggregated for a year, the individual could receive 0–8 for each behavior. We created GLMMs that included the behavior of interest (i.e. vocalization, movement or contact) as our response variable and either testing phase or trapping year as a fixed effect with raccoon ID as a random effect. We used these models to measure repeatability of each behavior by calculating intraclass correlation coefficients (ICCs) (Nakagawa and Schielzeth, 2010). Typically, ICC scores above 0.8 are considered to indicate high repeatability (Koo and Li, 2016). We also assessed repeatability in trappability using a similar GLMM with trappability score as our response variable.

#### Response to testing devices

Although the testing devices functioned as a source of food for the raccoons, the devices contained both visual (LED lights) and auditory stimuli (motor sound activated when reward was dispensed) that may have been perceived as risky by raccoons. Therefore, we wanted to understand whether and why raccoons exhibited different responses to the devices. We categorized all raccoons as having habituated (1) or not habituated (0) to the devices. Raccoons considered to have habituated to the device came to the device on multiple nights and ate their (three) free nightly rewards (i.e. readily fed from the boxes) and/or they learned the association between the buttons and the food reward (i.e. participated in reversal-learning trials; Movie 1). Raccoons that did not habituate to the device were those that approached the device and ran away as soon as the motor turned on, and either never returned or returned at some point but ran away again (i.e. never readily fed from the boxes; Movie 3). It is possible that some animals encountered the devices but were not close enough to be detected by the RFID antenna and were therefore not included in this study.

We expected that those raccoons that were bolder (i.e. less docile, more trappable) and/or were in a lower body condition would be more likely to habituate to the testing device. Because reactions to novelty and risk can be affected by the presence of conspecifics (Boogert et al., 2018; Moretti et al., 2015; Stöwe et al., 2006), we also expected that raccoons that first encountered the device alone may have been more likely to startle and ultimately not habituate to the device in comparison with those that encountered the device while with one or more conspecifics. To assess this possibility, we reviewed video footage of each raccoon's first encounter with the device and categorized each individual as being either alone (1) or not alone (0) when they first approached the device as our measure of sociality. We then examined whether and how our variables of interest predicted whether animals habituated to the devices (0,1) using binomial GLMs. We included the sex, age at time of testing and sociality of raccoons as fixed effects in our model, as well as any additional variables of interest that showed high repeatability (i.e. ICC>0.8). Because we expected that some of our measures might covary, we calculated generalized variance inflation factors (GVIFs) for all terms included in our final models (Fox and Monette, 1992).

Lastly, we examined whether and how our variables of interest predicted whether habituated individuals successfully learned to use the devices. We considered raccoons to have learned to use the testing device if they completed their training phase, as this required the raccoons to produce the learned behavior multiple times (i.e. push on the buttons 11 times). We did this by sub-setting our dataset to include only those individuals that habituated to the device. We expected that although all of these raccoons habituated to the devices, some individuals may have been more willing to enter and spend time in the device, which would facilitate better learning of the task. We also suspected that there may be some sex- or age-based differences in learning ability. We therefore categorized every individual to have learned (1) or not learned (0) how to use the devices. We then used GVIFs and binomial GLMs to test whether the fixed effects of sex, age at testing and repeatable measures of docility (e.g. vocalization, movement, contact) predicted a raccoon's success at learning to use the device.

#### Reversal learning in free-ranging raccoons

Using the same statistical methods outlined in Stanton et al. (2021), we measured performance in reversal learning by calculating the number of errors an individual made before reaching criterion (i.e. before completing a reversal). We first used GLMMs to test how reversal number, age, sex and prolonged breaks in testing time (i.e. the reversal started on one testing night but was completed on a separate, subsequent testing night; 0, 1) affected the number of errors a raccoon made with raccoon ID as a random effect. We then built separate GLMs for each individual with reversal number and correct side as fixed effects and number of errors as the response variable. This allowed us to determine whether individuals showed improvement by demonstrating a decreasing trend in number of errors made across reversals, and whether individuals had a side bias (i.e. significant effect of side assigned as the positive stimulus) that would affect performance.

#### Natural versus captive conditions

Finally, we compared the performance of our wild, free-ranging raccoons in natural conditions (described above) with that of the wild, captive raccoons tested in Stanton et al. (2021). We asked whether performance differed in natural versus captive conditions by testing the interaction between experimental condition and reversal number on the number of errors made using a GLMM, and included both random slopes and intercepts for raccoon ID. To investigate potential influence of age on reversal learning, we built a second GLMM of the same structure but removed juveniles from the dataset, which allowed us to compare adults from both natural and captive conditions. It is important to note that because our protocols differed in captive and natural conditions, we remain conservative in our inferences stemming from these comparisons.

### Ethical note

All trapping and experimental methods were approved under the University of Wyoming Institutional Animal Care and Use Committee (most recent protocol no. 20180813SB00321-02) and the Wyoming Game and Fish Department (Chapter 33 Permit ID: 1019), and are in accordance with the guidelines of the American Society of Mammologists for the use of wild mammals in research (Sikes, 2016). Research with captive raccoons was approved by the USDA National Wildlife Research Center Institute for Animal Care and Use Committee (QA-2825).

## RESULTS

### Biological and behavioral traits

From 2015 to 2019, we captured and marked 204 raccoons in Laramie: 25 adult females, 33 adult males, 67 juvenile females and 79 juvenile males. We found that juvenile raccoons were in poorer body condition compared with adults, and that female raccoons were in better body condition than males (Gaussian GLM: age: odds ratio=0.91, *P*<0.001; sex: odds ratio=0.94, *P*=0.01) (Table S2). *Post hoc* analyses revealed that the sex-based difference in BCS was driven by age, such that adult females differed from adult males (contrast test: *z*=−2.598, *P* =0.03), but juvenile females did not differ from juvenile males (contrast test: *z*=−0.070, *P*=0.99). We did not find any age- or sex-based differences in trappability (quasibinomial GLM: age: odds ratio=0.85, *P*=0.347; sex: odds ratio=1.17, *P*=0.391) (Table S2). The majority of raccoons were only captured/recaptured within a single trapping year; however, 25 individuals were captured in more than one trapping year between 2016 and 2019.

We collected 186 complete behavioral assessments from 165 individuals captured between 2016 and 2019. These 186 behavioral assessments were used to test repeatability of behaviors across the four testing phases. Testing phase had a significant effect on the expression of vocalizations and movement behavior within a trapping event (Table S3), indicating that the type of interaction with humans likely elicited different behavioral responses from the raccoons. Our model for contact with humans across testing phases was not supported, as contact with humans occurred very infrequently. We found moderate repeatability in vocalization (ICC=0.56) but no repeatability in movement (ICC=0.05) across testing phases. We collected two behavioral assessments for 20 of the 25 raccoons that were recaptured across trapping years, and we used these 40 assessments to calculate repeatability in vocalization, movement, and contact with humans across trapping years. Trapping year did not have an effect on any of the behaviors of interest (Table S3). We found high repeatability in vocalization (ICC=0.95), but no repeatability in movement (ICC=0.17) or contact (ICC=0.23). Therefore, we collected a single, aggregated vocalization score (0–7; interquartile range=3) taken from an individual's first behavioral assessment as an indicator of docility for that individual. Using this score, we did not detect any age- or sex-based difference in docility (cumulative link model: age: odds ratio=1.37, *P*=0.463; sex: odds ratio=2.14, *P*=0.102) (Table S2).

**Fig. 1.**
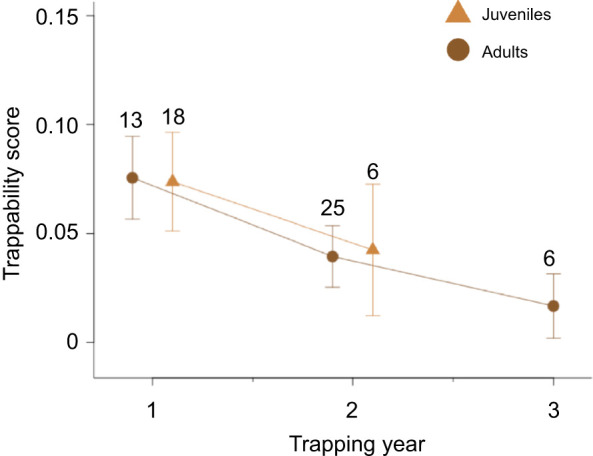
**Trappability scores for raccoons across trapping years (0=less trappable, 1=more trappable).** GLMs indicated that both adults and juveniles decreased in trappability from the first year they were trapped to the second year they were either trapped or not trapped but confirmed alive and on site via RFID detection. Numbers indicate sample size for each group. Raccoons over 2 years old are considered to be adults, which is why there are no data points for juveniles in trapping year 3 above.

We were able to calculate trappability scores for 31 individuals that were either captured or confirmed alive at a site following trapping efforts in 2016–2019, and used data from these 31 individuals to calculate repeatability in trappability. Trapping year had a significant, negative effect on trappability (GLMM: odds ratio=0.97, *P*=0.001) (Table S3), and we did not find repeatability in trappability (ICC=0.05). Further exploratory analyses of trappability, including the effects of age and sex, revealed that trappability decreased significantly from the first trapping year to the second trapping year (*post hoc* contrast test: *t=*−3.408, *P*=0.0029), but not from the second trapping year to the third (*post hoc* contrast test: *t=*−1.403, *P*=0.3013). This pattern was demonstrated by both juveniles and adults (*post hoc* contrast test: adults first observation to second observation: *t*=−3.408, *P*=0.0044; juveniles first observation to second observation: *t*=−3.408, *P*=0.0043), indicating that raccoons likely learned to avoid traps after their first trapping year, regardless of age (Fig. 1).

### Response to testing devices

**Fig. 2.**
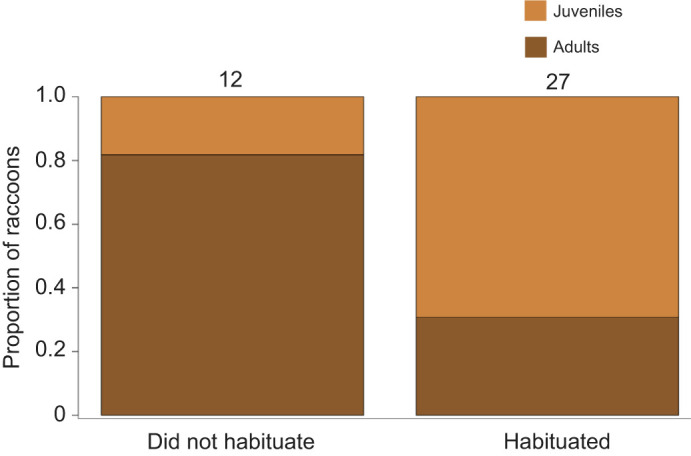
**Proportion of raccoons that did not habituate (left) or habituated (right) to the testing device.** Numbers represent sample size for each group. GLMs indicated that juveniles were more likely to habituate to the device than adults.

Our testing devices detected a total of 40 raccoons (14 adult females, 5 adult males, 12 juvenile females, 9 juvenile males), as well as four striped skunks (*Mephitis mephitis*; see Supplementary Materials and Methods for details), in 2018 and 2019. Of these 40 raccoons, 21 first encountered the device alone whereas 19 first encountered the device with one or more conspecifics. We found that juveniles were less likely to be alone upon their first encounter of the device compared with adults (binomial GLM: odds ratio=0.137, *P*=0.03) (Table S2). One raccoon (juvenile female) only encountered the device once on an early testing night when the device was malfunctioning, and was therefore removed from the dataset. Of the 39 remaining raccoons, 27 habituated to the device and 12 did not habituate to the device. Two raccoons lacked complete behavioral assessment data from when they were captured, and therefore data from 37 raccoons were used to evaluate predictors of habituation (Fig. 2). Because BCS and trappability were not repeatable and we lacked current scores for several of our participants, we included only age, sex, sociality and docility (i.e. first vocalization score) as predictors of habituation. None of the terms indicated covariance (GVIFs<3) and we used the dredge function and AICc model selection in MuMIn (https://CRAN.R-project.org/package=MuMIn) to compare all possible combinations of age, sex, sociality and docility. We performed full model averaging of two top models that were within 2 AICc values (Burnham and Anderson, 2002). Results from our averaged model indicated that juveniles were more likely to habituate to the device than adults, and that there was no influence of sex, sociality or docility (Table 1). Using the same statistical procedure with age, sex and docility as fixed effects (GVIFs<3), we found no effect of age or sex on learning to use the device. However, we found individuals with higher docility scores (i.e. lower vocalization scores) were more likely to learn to use the device than those with lower docility scores (i.e. higher vocalization scores) (Table 1).

**Table 1.**
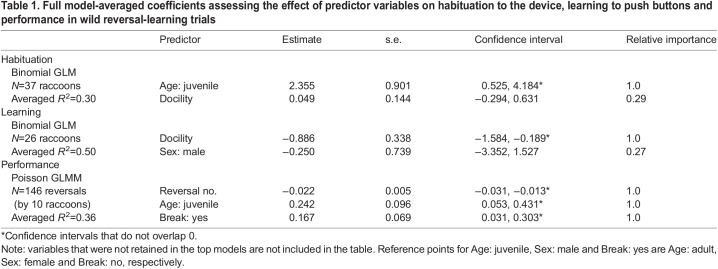
Full model-averaged coefficients assessing the effect of predictor variables on habituation to the device, learning to push buttons and performance in wild reversal-learning trials

### Reversal learning in free-ranging raccoons

Of the 27 raccoons that habituated to the device, 19 learned the association between pushing on the buttons and receiving a food reward and 17 completed serial reversals. However, we encountered a high amount of social interference, characterized by multiple raccoons attempting to enter the testing device at one time, resulting in erratic pushing of the buttons. Unfortunately, this meant that reversal-learning data were unreliable for seven of the 17 successful raccoons (five juveniles, two adults), leaving us with reversal-learning data for 10 raccoons.

Raccoons completed an average of 13 reversals (range: 3–38 reversals) and tended to make fewer errors across time (GLMM: odds ratio=0.98, *P*<0.0001) (Table 1). We also found that juveniles tended to make more errors than adults (GLMM: odds ratio=1.27, *P*=0.01), and that an overnight break in testing resulted in a higher number of errors for that reversal compared with reversals that were completed within a single testing night (GLMM: odds ratio=1.18, *P*=0.02) (Table 1). Three raccoons showed significant improvement across serial reversals, and an additional four raccoons also showed a trend towards improvement characterized by a negative slope (Table 2; Table S4). In addition, it appeared that four raccoons likely had a right-side bias (Table 2).

**Table 2.**
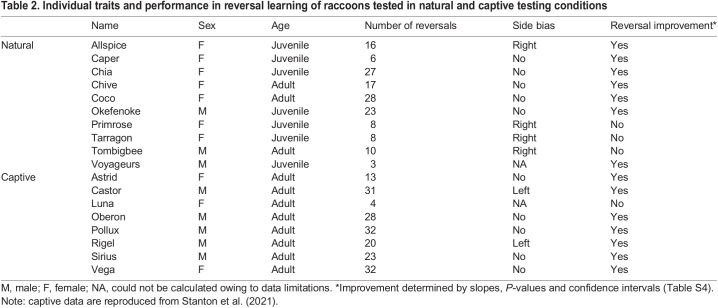
Individual traits and performance in reversal learning of raccoons tested in natural and captive testing conditions

### Natural versus captive conditions

**Fig. 3.**
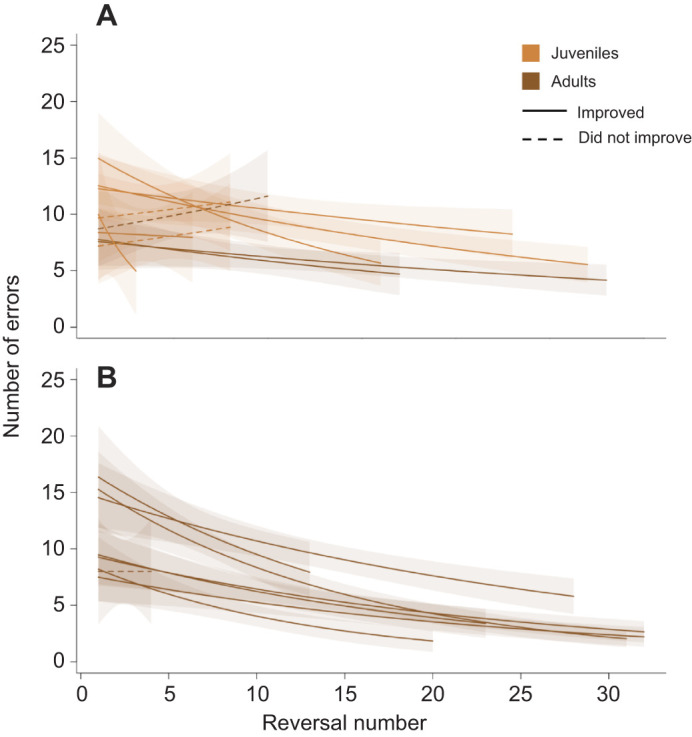
**Learning curves (±s.e.) for each study participant based on generalized linear models of individual performance.** Raccoons were tested in (A) natural and (B) captive conditions. Individuals in both conditions generally demonstrated learning of this task; however, there is variation in performance. Solid lines represent individuals that showed improvement by making fewer errors across reversals, whereas dashed lines indicate that the individual did not show improvement across reversals. AICc model selection indicated that adults made fewer errors compared with juveniles in natural conditions. Although our results suggest that wild raccoons tested in captive conditions (B) outperformed wild raccoons tested in natural environments (A), it should be noted that protocols differed slightly between the two environments.

Raccoons tested in captivity generally completed more reversals (mean=27, range=4–32) than raccoons tested in natural conditions (mean=13, range=3–28). Comparisons of performance between the two groups (Fig. 3) suggested that raccoons did not differ in the number of errors being made initially (i.e. did not differ in intercept), but did demonstrate a difference in the number of errors being made across reversal events (i.e. differed in slope) (Table S5). Specifically, raccoons tested in captivity decreased the numbers of errors being made at a faster rate across reversals (GLMM: odds ratio=1.023, *P*=0.004), and this remained constant when juveniles were removed from the dataset (GLMM: odds ratio=1.029, *P*=0.02).

## DISCUSSION

Animals living in anthropogenic landscapes are faced with both risks and rewards associated with humans (Schell et al., 2021). One strategy believed to facilitate adaptation to anthropogenic landscapes is use of cognition, which can allow animals to behave flexibly and thereby exploit or avoid various opportunities and threats (Sol et al., 2013). Here, we delivered a classic test of cognitive flexibility to a wild population of raccoons – a successful urban exploiter known for its adaptability and cognitive aptitude. Although most raccoons habituated to the testing device and many learned to complete serial reversals, we found that biological and behavioral factors may have influenced participation. Furthermore, we encountered some extraneous challenges associated with testing in natural conditions that likely influenced performance in reversal learning as well.

### Response to testing and participation

First, we were interested in how motivation to participate may vary among sexes and age classes. We found that juvenile raccoons tended to be in poorer body condition than adults, and adult females tended to be in better body condition than adult males. This pattern is not surprising; males and females in sexually dimorphic species, such as raccoons, have different energetic demands (Isaac, 2005), and the difference in body condition between age classes is typically greater than within age classes in raccoons (Gehrt, 2003). Adult male and female raccoons also differ in their degree of sociality: adult males tend to associate more with other adult males, whereas adult females have more ephemeral associations but are often accompanied by kits (i.e. young juveniles) during the summer (Prange et al., 2011). Although we have observed similar associations in our Laramie population, we have also observed that as kits begin to gain independence in late summer through autumn, they often travel and forage with other kits (R. Fanelli, L.A.S., D. McDonald and S.B.-A., unpublished data). These associations among kits likely explain why we found that juveniles were less likely to be alone upon their first encounter with the testing device. We therefore believe that energetic demands and sociality, both of which appear higher in juveniles in our population, could explain why juveniles were more likely to habituate to the testing devices than adults. In addition, juveniles are less experienced and often more exploratory than adults, given a greater need to gather information about their environment in early life (Greenberg and Mettke-Hofmann, 2001; Reader, 2015). Thus, it is possible that their naïveté and greater exploratory tendencies also facilitated habituation. However, juveniles were not more trappable than adults, indicating that they can learn to avoid adverse situations. Therefore, our observations suggest that juvenile and adult raccoons will discriminate between risky and profitable situations, but that the behavioral and biological traits typical of juveniles may increase the likelihood of exploitation of profitable opportunities.

In accordance with previous research (e.g. Debeffe et al., 2015; Petelle et al., 2013), we found that individual variation in docility was highly repeatable across trapping years. We also found that raccoons that demonstrated greater docility towards humans were more likely to learn how the testing device operated. This pattern might be explained by a relationship between emotional reactivity and cognitive ability (e.g. emotional reactivity hypothesis; Hare and Tomasello, 2005), which may be particularly relevant in urban environments. For example, the increased frequency of human–wildlife interactions via urbanization may be imposing selection pressures similar to that of domestication, whereby animals become less aggressive and responsive towards some environmental stimuli (e.g. via altered stress responses) (Geffroy et al., 2015, 2020a). A large body of literature suggests that the process of domestication also yields changes in cognitive ability, including, in some cases, improved learning and flexibility in behavior (e.g. Hare, 2017; Hare et al., 2002; Lewejohann et al., 2010; but see Range and Marshall-Pescini, 2022). Similarly, growing evidence suggests that shy, less aggressive and less active animals represent a reactive phenotype characterized by greater flexibility, as well as low speed–high accuracy trade-offs in cognition (Bray et al., 2015; Koolhaas et al., 2010; Sih and Del Giudice, 2012). Reactive phenotypes may be favored in urban environments, whereby individuals that are able to cope with the presence of humans and novel stimuli will be more likely to persist via increased learning and flexibility (Geffroy et al., 2020b). Therefore, future research on raccoons and other urban species would likely benefit from specific investigation of the link between docility towards humans and cognitive ability, as we suspect this may influence important aspects of wildlife behavior, including domestication and nuisance-prone activities (see below).

Although the presence of conspecifics did not directly predict habituation to the device in our analyses, social factors certainly played a prominent role in this study. We observed an unexpectedly high amount of social interference during trials. At times, raccoons demonstrated aggression towards one another, resulting in displacement from the testing device. At other times, raccoons were more tolerant of one another and shared access to the device, which resulted in the erratic pushing of buttons and ultimately led to the removal of data for seven individuals. Removal of these data was necessary to ensure the quality and interpretation of cognitive performance; however, we recognize that our reversal-learning dataset may be biased towards more asocial individuals, or perhaps those that were better able to maintain access to the device (i.e. more dominant individuals that are less likely to be displaced). Although group composition and sociality may indeed influence the development and evolution of cognition (e.g. Aplin and Morand-Ferron, 2017; Ashton et al., 2018; Dunbar and Shultz, 2007), it is currently difficult to speculate on whether and how the differences in sociality observed in our study reflect actual differences in cognitive ability. Further research is currently needed to understand not only how raccoon sociality relates to their cognition in general, but also how raccoon social behavior and cognition may be changing as a result of the increasing densities and aggregations of raccoons in urban environments (Hauver et al., 2013; Schuttler et al., 2015; Wehtje and Gompper, 2011).

### Reversal-learning performance

With regard to performance in reversal learning, we found that raccoons tested in natural conditions showed a general decrease in the number of errors being made across serial reversals, but that this was sensitive to prolonged gaps in testing (as is typical in studies of reversal learning; e.g. Mackintosh et al., 1968). Furthermore, our analyses suggest that juveniles made a higher number of errors than adults, which may be due to developmental effects (Kayser et al., 2021) or perhaps the ability of juveniles to maintain undisturbed access to the device (Hauver et al., 2013). It should be noted, however, that many juveniles completed a high number of reversals and showed improvement over time. In addition, juvenile raccoons have mastered tasks requiring learning and have demonstrated flexible problem-solving abilities (e.g. Stanton et al., 2017). Thus, given our limited sample size, this difference in performance between juveniles and adults should be interpreted with caution.

We also found that wild raccoons tested in natural conditions completed fewer reversals and improved at a slower rate than wild raccoons tested in captive conditions. This pattern persisted even after reversal-learning data for juveniles were removed, indicating that this divergence was unlikely to have stemmed from potential differences between juveniles and adults. Unlike raccoons tested in captivity, raccoons tested in natural conditions had a greater need for vigilance and were observed frequently exiting the testing device to scan their surroundings (Movie 1). Moreover, captive raccoons received concentrated, undisturbed testing sessions, whereas testing in natural conditions was less structured and more susceptible to perturbations. Such distractions have been implicated in other cognitive studies comparing captive versus wild populations of carnivores (e.g. Benson-Amram et al., 2013), and likely contributed to greater performance by raccoons in captive conditions. Nevertheless, raccoons in both groups demonstrated rapid acquisition of this task and an ability to form and reverse learned associations, which is indicative of cognitive flexibility.

### Cognition in the field

Our study presents several implications for the study of cognition in the field, as well as our more general understanding of nuisance animal cognition. First, in our efforts to adapt a classic cognitive paradigm (i.e. reversal learning) for assessment of large-bodied, terrestrial organisms, we highlight some of the challenges that persist when testing such non-traditional species in the field. For example, it appears that, unlike other studies focused on birds (e.g. Cauchoix et al., 2017), the lack of control inherent to field testing (e.g. prolonged gaps in testing, multiple individuals present) influenced our ability to collect data on reversal-learning performance for all raccoons tested. Furthermore, we point out that social interference may be especially difficult to overcome in anthropogenic landscapes, where increased gregariousness may be more common in certain taxa. Therefore, researchers interested in similar investigations must be prepared to circumvent heightened vigilance and social interference, perhaps by adjusting learning criteria, shortening testing periods and/or creating automated devices that only allow admittance of one individual at a time (e.g. Muns et al., 2018).

Second, we identified several biological and behavioral traits that may contribute to individual variation in participation and performance in our study. Representation of all four age–sex classes within our dataset paired with the individual variation we encountered during behavioral assessments and cognitive testing (e.g. vocalization range=0–7; interquartile range=3) suggests that our trapping and identification efforts are not limited to one specific behavioral phenotype, despite raccoons learning to avoid being trapped over time. This is essential not only for reducing sampling biases, but also for understanding individual variation in cognitive ability (Boogert et al., 2018; Webster and Rutz, 2020). Given that docility was repeatable and had implications for raccoon learning ability in our study, we believe that vocalization is a promising behavioral measure for use in future studies on individual variation in raccoons. Furthermore, vocalizations could be used to assess interpopulation and intrapopulation docility towards humans, which may reveal how anthropogenic features, including human behavior, drive local adaptation and the possible domestication of urban wildlife.

Lastly, we suggest that a greater understanding of individual variation in species perceived as a nuisance will bolster our ability to mitigate human–wildlife conflict. Simple forms of learning, such as habituation and sensitization, as well as flexibility in behavior, often undermine non-lethal mitigation strategies such as playbacks, effigies and exclusion structures (Barrett et al., 2019; Blumstein, 2016). Therefore, identifying the biological and behavioral traits that influence learning, flexibility and risk-taking is essential for coexistence in expanding anthropogenic landscapes. Based on our results, managers and members of the public may need to be particularly aware of learning in juvenile raccoons, and therefore might consider focusing humane mitigation efforts (e.g. aversive conditioning) during times when juveniles are first venturing out of dens. Although our sample size is limited, we present evidence that raccoons quickly learn to avoid risky or adverse situations (e.g. trapping), but are able to exploit benign, profitable opportunities (e.g. feeding from an automated device). This discrimination ability and selectivity in risk-taking is likely to aid their successful adaptation to anthropogenic areas, but whether such learned risk-perception and flexibility is ubiquitous across raccoon populations or other urban species is an open question. Finally, based on our observations of wild raccoons, we see the potential for greater integration of animal personality research into studies of nuisance animal cognition. For example, problem individuals are often cited for their boldness because of a willingness to engage with novelty and incur risks (Barrett et al., 2019). Although bolder, proactive individuals may indeed be prone to more obvious forms of conflict (e.g. approaching humans), it is possible that shyer, reactive individuals are prone to less obvious forms of conflict that require greater associative learning or flexibility in behavior (e.g. raiding chicken coops). In this way, lethal removal of proactive individuals may be artificially selecting for reactive and particularly capable individuals that ultimately represent a greater management challenge (Schell et al., 2021).

### Conclusions

In summary, our investigation has identified several environmental, individual and social factors that influence raccoon behavior and performance in cognitive testing. These results provide important insights into the ecology of raccoons, as well as our ability to study the contemporary evolution of cognition and behavior. We hope that this study will serve as an important step in expanding tests of cognition to non-traditional species and further research on the cognition of, and coexistence with, urban wildlife.

## Supplementary Material

10.1242/jexbio.243726_sup1 Supplementary information

## References

[JEB243726C1] Allman, J., McLaughlin, T. and Hakeem, A. (1993). Brain weight and life-span in primate species. *Proc. Natl. Acad. Sci. USA* 90, 118-122. 10.1073/pnas.90.1.118 8419913 PMC45611

[JEB243726C2] Amiel, J. J., Tingley, R. and Shine, R. (2011). Smart moves: effects of relative brain size on establishment success of invasive amphibians and reptiles. *PLoS One* 6, e18277. 10.1371/journal.pone.0018277 21494328 PMC3071803

[JEB243726C3] Aplin, L. M. and Morand-Ferron, J. (2017). Stable producer–scrounger dynamics in wild birds: sociability and learning speed covary with scrounging behaviour. *Proc. R. Soc. B Biol. Sci.* 284, 20162872. 10.1098/rspb.2016.2872 PMC5394662 28404775

[JEB243726C4] Aplin, L. M., Farine, D. R., Morand-Ferron, J., Cockburn, A., Thornton, A. and Sheldon, B. C. (2015). Experimentally induced innovations lead to persistent culture via conformity in wild birds. *Nature* 518, 538-541. 10.1038/nature13998 25470065 PMC4344839

[JEB243726C5] Ashton, B. J., Ridley, A. R., Edwards, E. K. and Thornton, A. (2018). Cognitive performance is linked to group size and affects fitness in Australian magpies. *Nature* 554, 364-367. 10.1038/nature25503 29414945 PMC5815499

[JEB243726C6] Audet, J.-N. and Lefebvre, L. (2017). What's flexible in behavioral flexibility? *Behav. Ecol.* 28, 943-947. 10.1093/beheco/arx007

[JEB243726C7] Audet, J.-N., Ducatez, S. and Lefebvre, L. (2016). The town bird and the country bird: problem solving and immunocompetence vary with urbanization. *Behav. Ecol.* 27, 637-644. 10.1093/beheco/arv201

[JEB243726C8] Audubon, J. and Bachman, J. (1851). *The Quadrupeds of North America Volume II*. New York: V. G. Audubon.

[JEB243726C9] Barrett, L. P., Stanton, L. and Benson-Amram, S. (2019). The cognition of ‘nuisance’ species. *Anim. Behav.* 147, 167-177. 10.1016/j.anbehav.2018.05.005

[JEB243726C10] Benson-Amram, S., Weldele, M. L. and Holekamp, K. E. (2013). A comparison of innovative problem-solving abilities between wild and captive spotted hyaenas, *Crocuta crocuta*. *Anim. Behav.* 85, 349-356. 10.1016/j.anbehav.2012.11.003

[JEB243726C11] Blumstein, D. T. (2016). Habituation and sensitization: new thoughts about old ideas. *Anim. Behav.* 120, 255-262. 10.1016/j.anbehav.2016.05.012

[JEB243726C12] Boogert, N. J., Madden, J. R., Morand-Ferron, J. and Thornton, A. (2018). Measuring and understanding individual differences in cognition. *Philos. Trans. R. Soc. B Biol. Sci.* 373, 20170280. 10.1098/rstb.2017.0280 PMC6107562 30104425

[JEB243726C13] Bray, E. E., MacLean, E. L. and Hare, B. A. (2015). Increasing arousal enhances inhibitory control in calm but not excitable dogs. *Anim. Cogn.* 18, 1317-1329. 10.1007/s10071-015-0901-1 26169659 PMC4609265

[JEB243726C14] Bridge, E. S., Wilhelm, J., Pandit, M. M., Moreno, A., Curry, C. M., Pearson, T. D., Proppe, D. S., Holwerda, C., Eadie, J. M., Stair, T. F. et al. (2019). An Arduino-based RFID platform for animal research. *Front. Ecol. Evol.* 7, 257. 10.3389/fevo.2019.00257

[JEB243726C95] Burnham, K. P. and Anderson, D. R. (2002) Model Selection and Multimodel Inference. *A Practical Information–Theoretic Approach, 2nd edn. Springer*. 10.1007/b97636

[JEB243726C15] Carrete, M., Tella, J. L. (2011). Inter-individual variability in fear of humans and relative brain size of the species are related to contemporary urban invasion in birds. *PLoS One* 6, e18859. 10.1371/journal.pone.0018859 21526193 PMC3079730

[JEB243726C16] Carter, A. J., Heinsohn, R., Goldizen, A. W. and Biro, P. A. (2012). Boldness, trappability and sampling bias in wild lizards. *Anim. Behav.* 83, 1051-1058. 10.1016/j.anbehav.2012.01.033

[JEB243726C17] Cauchoix, M., Hermer, E., Chaine, A. S. and Morand-Ferron, J. (2017). Cognition in the field: comparison of reversal learning performance in captive and wild passerines. *Sci. Rep.* 7, 12945. 10.1038/s41598-017-13179-5 29021558 PMC5636806

[JEB243726C96] Chow, P. K. Y., Clayton, N. S. and Steele, M. A. (2021). Cognitive performance of wild eastern gray squirrels (Sciurus carolinensis) in rural and urban, native, and non-native environments. *Frontiers in Ecology and Evolution* 9, 615899. 10.3389/fevo.2021.615899

[JEB243726C18] Daniels, S. E., Fanelli, R. E., Gilbert, A. and Benson-Amram, S. (2019). Behavioral flexibility of a generalist carnivore. *Anim. Cogn.* 22, 387-396. 10.1007/s10071-019-01252-7 30805799

[JEB243726C19] Debeffe, L., Lemaître, J. F., Bergvall, U. A., Hewison, A. J. M., Gaillard, J. M., Morellet, N., Goulard, M., Monestier, C., David, M., Verheyden-Tixier, H. et al. (2015). Short- and long-term repeatability of docility in the roe deer: sex and age matter. *Anim. Behav.* 109, 53-63. 10.1016/j.anbehav.2015.08.003

[JEB243726C20] Dunbar, R. I. M. and Shultz, S. (2007). Evolution in the social brain. *Science* 317, 1344-1347. 10.1126/science.1145463 17823343

[JEB243726C22] Fox, J. and Monette, G. (1992). Generalized collinearity diagnostics. *J. Am. Stat. Assoc.* 87, 178-183. 10.1080/01621459.1992.10475190

[JEB243726C23] Garamszegi, L. Z., Eens, M. and Török, J. (2009). Behavioural syndromes and trappability in free-living collared flycatchers, *Ficedula albicollis*. *Anim. Behav.* 77, 803-812. 10.1016/j.anbehav.2008.12.012

[JEB243726C24] Geffroy, B., Samia, D. S. M., Bessa, E. and Blumstein, D. T. (2015). How nature-based tourism might increase prey vulnerability to predators. *Trends Ecol. Evol.* 30, 755-765. 10.1016/j.tree.2015.09.010 26475119

[JEB243726C25] Geffroy, B., Sadoul, B., Putman, B. J., Berger-Tal, O., Garamszegi, L. Z., Møller, A. P. and Blumstein, D. T. (2020a). Evolutionary dynamics in the anthropocene: life history and intensity of human contact shape antipredator responses. *PLoS Biol.* 18, e3000818. 10.1371/journal.pbio.3000818 32960897 PMC7508406

[JEB243726C26] Geffroy, B., Alfonso, S., Sadoul, B. and Blumstein, D. T. (2020b). A world for reactive phenotypes. *Front. Conserv. Sci.* 1, 611919. 10.3389/fcosc.2020.611919

[JEB243726C27] Gehrt, S. D. (2003). Raccoons and allies. In *Wild Mammals of North America: Biology, Management, and Conservation* (ed. G. A. Feldhamer, B. C. Thompson and J. A. Chapman), pp. 611-633. Baltimore: Johns Hopkins University Press.

[JEB243726C28] Gehrt, S. D. (2004). Ecology and management of striped skunks, raccoons, and coyotes in urban landscapes. In *People and Predators: From Conflict to Coexistence* (ed. N. Fascione, A. Delach and M. Smith), pp. 81-104. Island Press.

[JEB243726C29] Ginsburg, S. and Jablonka, E. (2010). The evolution of associative learning: a factor in the Cambrian explosion. *J. Theor. Biol.* 266, 11-20. 10.1016/j.jtbi.2010.06.017 20558182

[JEB243726C30] Grau, G. A., Sanderson, G. C. and Rogers, J. P. (1970). Age Determination of Raccoons. *The Journal of Wildlife Management* 34, 364-372. 10.2307/3799023.

[JEB243726C31] Greenberg, R. and Mettke-Hofmann, C. (2001). Ecological aspects of neophobia and neophilia in birds. In *Current Ornithology*, Vol. 16 (ed. V. Nolan, C. F. Thompson), pp. 119-178. Boston: Springer. 10.1007/978-1-4615-1211-0_3

[JEB243726C32] Griebling, H. J., Sluka, C. M., Stanton, L. A., Barrett, L. P., Bastos, J. B. and Benson-Amram, S. (2022). How technology can advance the study of animal cognition in the wild. *Curr. Opin. Behav. Sci.* 45, 101120. 10.1016/j.cobeha.2022.101120

[JEB243726C33] Griffin, A. S., Netto, K. and Peneaux, C. (2017). Neophilia, innovation and learning in an urbanized world: a critical evaluation of mixed findings. *Curr. Opin. Behav. Sci.* 16, 15-22. 10.1016/j.cobeha.2017.01.004

[JEB243726C34] Hadidian, J., Prange, S., Rosatte, R., Riley, S. and Gehrt, S. (2010). Raccoons (Procyon lotor). In *Urban Carnivores: Ecology, Conflict and Conservation* (ed. S. D. Gehrt, S. P. D. Riley and B. L. Cypher), pp. 35-47. Baltimore: JHU Press.

[JEB243726C35] Hare, B. (2017). Survival of the friendliest: *Homo sapiens* evolved via selection for prosociality. *Annu. Rev. Psychol.* 68, 155-186. 10.1146/annurev-psych-010416-044201 27732802

[JEB243726C36] Hare, B. and Tomasello, M. (2005). Human-like social skills in dogs? *Trends Cogn. Sci.* 9, 439-444. 10.1016/j.tics.2005.07.003 16061417

[JEB243726C37] Hare, B., Brown, M., Williamson, C. and Tomasello, M. (2002). The domestication of social cognition in dogs. *Science* 298, 1634-1636. 10.1126/science.1072702 12446914

[JEB243726C38] Hauver, S., Hirsch, B. T., Prange, S., Dubach, J. and Gehrt, S. D. (2013). Age, but not sex or genetic relatedness, shapes raccoon dominance patterns. *Ethology* 119, 769-778. 10.1111/eth.12118

[JEB243726C39] Isaac, J. L. (2005). Potential causes and life-history consequences of sexual size dimorphism in mammals. *Mamm. Rev.* 35, 101-115. 10.1111/j.1365-2907.2005.00045.x

[JEB243726C40] Izquierdo, A., Brigman, J. L., Radke, A. K., Rudebeck, P. H. and Holmes, A. A. (2017). Review the neural basis of reversal learning: an updated perspective. *Neuroscience* 345, 12-26. 10.1016/j.neuroscience.2016.03.021 26979052 PMC5018909

[JEB243726C41] Jacob, J., Kent, M., Benson-Amram, S., Herculano-Houzel, S., Raghanti, M. A., Ploppert, E., Drake, J., Hindi, B., Natale, N. R., Daniels, S. et al. (2021). Cytoarchitectural characteristics associated with cognitive flexibility in raccoons. *J. Comp. Neurol.* 529, 3375-3388. 10.1002/cne.25197 34076254

[JEB243726C42] Jardim-Messeder, D., Lambert, K., Noctor, S., Pestana, F. M., de Castro Leal, M. E., Bertelsen, M. F., Alagaili, A. N., Mohammad, O. B., Manger, P. R. and Herculano-Houzel, S. (2017). Dogs have the most neurons, though not the largest brain: trade-off between body mass and number of neurons in the cerebral cortex of large carnivoran species. *Front. Neuroanat.* 11, 118. 10.3389/fnana.2017.00118 29311850 PMC5733047

[JEB243726C43] Johnson-Ulrich, L., Yirga, G., Strong, R. L. and Holekamp, K. E. (2021). The effect of urbanization on innovation in spotted hyenas. *Anim. Cogn.* 24, 1027-1038. 10.1007/s10071-021-01494-4 33687598

[JEB243726C44] Justice, D. H. (2021). *Raccoon*. Reaktion Books.

[JEB243726C45] Kayser, A. S., Schmidt, J. R., Chase, H. W., Weiss, E. O., Kruppa, J. A., Fink, G. R., Herpertz-Dahlmann, B., Konrad, K. and Schulte-Rüther, M. (2021). Developmental differences in probabilistic reversal learning: a computational modeling approach. *Front. Neurosci*. 14, 536596. 10.3389/fnins.2020.536596 33536865 PMC7848134

[JEB243726C46] Koo, T. K. and Li, M. Y. (2016). A guideline of selecting and reporting intraclass correlation coefficients for reliability research. *J. Chiropr. Med.* 15, 155-163. 10.1016/j.jcm.2016.02.012 27330520 PMC4913118

[JEB243726C47] Koolhaas, J. M., de Boer, S. F., Coppens, C. M. and Buwalda, B. (2010). Neuroendocrinology of coping styles: towards understanding the biology of individual variation. *Front. Neuroendocrinol.* 31, 307-321. 10.1016/j.yfrne.2010.04.001 20382177

[JEB243726C48] Lambert, M. R., Brans, K. I., Des Roches, S., Donihue, C. M. and Diamond, S. E. (2021). Adaptive evolution in cities: progress and misconceptions. *Trends Ecol. Evol.* 36, 239-257. 10.1016/j.tree.2020.11.002 33342595

[JEB243726C49] Lea, S. E. G., Chow, P. K. Y., Leaver, L. A. and McLaren, I. P. L. (2020). Behavioral flexibility: a review, a model, and some exploratory tests. *Learn. Behav.* 48, 173-187. 10.3758/s13420-020-00421-w 32043268 PMC7082303

[JEB243726C50] Lee, V. E. and Thornton, A. (2021). Animal cognition in an urbanised world. *Front. Ecol. Evol.* 9, 633947. 10.3389/fevo.2021.633947 34409044 PMC7611524

[JEB243726C51] Lewejohann, L., Pickel, T., Sachser, N. and Kaiser, S. (2010). Wild genius - domestic fool? Spatial learning abilities of wild and domestic guinea pigs. *Front. Zool.* 7, 9. 10.1186/1742-9994-7-9 20334697 PMC2859863

[JEB243726C52] Louppe, V., Leroy, B., Herrel, A. and Veron, G. (2019). Current and future climatic regions favourable for a globally introduced wild carnivore, the raccoon *Procyon lotor*. *Sci. Rep.* 9, 9174. 10.1038/s41598-019-45713-y 31235806 PMC6591328

[JEB243726C53] Mackintosh, N. J., Mcgonigle, B. and Holgate, V. (1968). Factors underlying improvement in serial reversal learning. *Can. J. Psychol. Can. Psychol.* 22, 85-95. 10.1037/h0082753 5649040

[JEB243726C54] Mettke-Hofmann, C. (2014). Cognitive ecology: ecological factors, life-styles, and cognition. *Wiley Interdiscip. Rev. Cogn. Sci.* 5, 345-360. 10.1002/wcs.1289 26308568

[JEB243726C55] Moll, R. J., Cepek, J. D., Lorch, P. D., Dennis, P. M., Tans, E., Robison, T., Millspaugh, J. J. and Montgomery, R. A. (2019). What does urbanization actually mean? A framework for urban metrics in wildlife research. *J. Appl. Ecol.* 56, 1289-1300. 10.1111/1365-2664.13358

[JEB243726C56] Morand-Ferron, J. (2017). Why learn? The adaptive value of associative learning in wild populations. *Curr. Opin. Behav. Sci.* 16, 73-79. 10.1016/j.cobeha.2017.03.008

[JEB243726C57] Morand-Ferron, J., Cole, E. F. and Quinn, J. L. (2015a). Studying the evolutionary ecology of cognition in the wild: a review of practical and conceptual challenges. *Biol. Rev.* 91, 367-389. 10.1111/brv.12174 25631282

[JEB243726C58] Morand-Ferron, J., Hamblin, S., Cole, E. F., Aplin, L. M. and Quinn, J. L. (2015b). Taking the operant paradigm into the field: associative learning in wild great tits. *PLoS One* 10, e0133821. 10.1371/journal.pone.0133821 26288131 PMC4546055

[JEB243726C59] Moretti, L., Hentrup, M., Kotrschal, K. and Range, F. (2015). The influence of relationships on neophobia and exploration in wolves and dogs. *Anim. Behav.* 107, 159-173. 10.1016/j.anbehav.2015.06.008 26405301 PMC4550430

[JEB243726C60] Muns, S. J., Hoy, J. M. and Murray, P. J. (2018). Microchips for macropods: first use of a microchip-automated door by a bridled nailtail wallaby (*Onychogalea fraenata*). *Zoo Biol.* 37, 274-278. 10.1002/zoo.21419 29923213

[JEB243726C61] Nakagawa, S. and Schielzeth, H. (2010). Repeatability for Gaussian and non-Gaussian data: a practical guide for biologists. *Biol. Rev.* 85, 935-956. 10.1111/j.1469-185X.2010.00141.x 20569253

[JEB243726C62] Pacini-Ketchabaw, V. and Nxumalo, F. (2015). Unruly raccoons and troubled educators: nature/culture divides in a childcare centre. *Environ. Humanit.* 7, 151-168. 10.1215/22011919-3616380

[JEB243726C63] Papini, M. R. (2002). Pattern and process in the evolution of learning. *Psychol. Rev.* 109, 186-201. 10.1037/0033-295X.109.1.186 11863037

[JEB243726C64] Petelle, M. B., McCoy, D. E., Alejandro, V., Martin, J. G. A. and Blumstein, D. T. (2013). Development of boldness and docility in yellow-bellied marmots. *Anim. Behav.* 86, 1147-1154. 10.1016/j.anbehav.2013.09.016

[JEB243726C65] Pitt, J. A., Larivière, S. and Messier, F. (2008). Survival and body condition of raccoons at the edge of the range. *J. Wildl. Manage.* 72, 389-395. 10.2193/2005-761

[JEB243726C66] Prange, S., Gehrt, S. D. and Hauver, S. (2011). Frequency and duration of contacts between free-ranging raccoons: uncovering a hidden social system. *J. Mammal.* 92, 1331-1342. 10.1644/10-MAMM-A-416.1

[JEB243726C67] Range, F. and Marshall-Pescini, S. (2022). Comparing wolves and dogs: current status and implications for human ‘self-domestication’. *Trends Cogn. Sci.* 26, 337-349. 10.1016/j.tics.2022.01.003 35294857

[JEB243726C68] Reader, S. M. (2015). Causes of individual differences in animal exploration and search. *Top. Cogn. Sci.* 7, 451-468. 10.1111/tops.12148 25982255

[JEB243726C69] Réale, D., Gallant, B. Y., Leblanc, M. and Festa-Bianchet, M. (2000). Consistency of temperament in bighorn ewes and correlates with behaviour and life history. *Anim. Behav.* 60, 589-597. 10.1006/anbe.2000.1530 11082229

[JEB243726C70] Sayol, F., Sol, D. and Pigot, A. L. (2020). Brain size and life history interact to predict urban tolerance in birds. *Front. Ecol. Evol.* 8, 58. 10.3389/fevo.2020.00058

[JEB243726C71] Schell, C. J., Stanton, L. A., Young, J. K., Angeloni, L. M., Lambert, J. E., Breck, S. W. and Murray, M. H. (2021). The evolutionary consequences of human–wildlife conflict in cities. *Evol. Appl.* 14, 178-197. 10.1111/eva.13131 33519964 PMC7819564

[JEB243726C72] Schulte-Hostedde, A. I., Zinner, B., Millar, J. S. and Hickling, G. J. (2005). Restitution of mass-size residuals: validating body condition indices. *Ecology* 86, 155-163. 10.1890/04-0232

[JEB243726C73] Schuttler, S. G., Ruiz-López, M. J., Monello, R., Wehtje, M., Eggert, L. S. and Gompper, M. E. (2015). The interplay between clumped resources, social aggregation, and genetic relatedness in the raccoon. *Mammal Res.* 60, 365-373. 10.1007/s13364-015-0231-3

[JEB243726C74] Shettleworth, S. J. (2010). *Cognition, Evolution, and Behaviour*, 2nd edn. New York: Oxford University Press.

[JEB243726C75] Sieber, O. J. (1984). Vocal communication in raccoons (*Procyon lotor*). *Behaviour* 90, 80-113. 10.1163/156853984X00560

[JEB243726C76] Sih, A. and Del Giudice, M. (2012). Linking behavioural syndromes and cognition: a behavioural ecology perspective. *Philos. Trans. R. Soc. Lond. B. Biol. Sci.* 367, 2762-2772. 10.1098/rstb.2012.0216 22927575 PMC3427552

[JEB243726C77] Sikes, R. S. (2016). 2016 guidelines of the American Society of Mammalogists for the use of wild mammals in research and education. *J. Mammal.* 97, 663-688. 10.1093/jmammal/gyw078 29692469 PMC5909806

[JEB243726C78] Skinner, B. F. (1938). *The Behavior of Organisms: An Experimental Analysis*. New York: Appleton-Century.

[JEB243726C79] Sol, D. (2009). Revisiting the cognitive buffer hypothesis for the evolution of large brains. *Biol. Lett.* 5, 130-133. 10.1098/rsbl.2008.0621 19049952 PMC2657766

[JEB243726C80] Sol, D., Duncan, R. P., Blackburn, T. M., Cassey, P. and Lefebvre, L. (2005). Big brains, enhanced cognition, and response of birds to novel environments. *Proc. Natl. Acad. Sci. USA* 102, 5460-5465. 10.1073/pnas.0408145102 15784743 PMC556234

[JEB243726C81] Sol, D., Bacher, S., Reader, S. M. and Lefebvre, L. (2008). Brain size predicts the success of mammal species introduced into novel environments. *Am. Nat.* 172, S63-S71. 10.1086/588304 18554145

[JEB243726C82] Sol, D., Lapiedra, O. and Gonzalezlez-Lagos, C. (2013). Behavioural adjustments for a life in the city. *Anim. Behav.* 85, 1101-1112. 10.1016/j.anbehav.2013.01.023

[JEB243726C83] Stanton, L., Davis, E., Johnson, S., Gilbert, A. and Benson-Amram, S. (2017). Adaptation of the Aesop's Fable paradigm for use with raccoons (*Procyon lotor*): considerations for future application in non-avian and non-primate species. *Anim. Cogn.* 20, 1147-1152. 10.1007/s10071-017-1129-z 28963599

[JEB243726C84] Stanton, L. A., Bridge, E. S., Huizinga, J., Johnson, S. R., Young, J. K. and Benson-Amram, S. (2021). Variation in reversal learning by three generalist mesocarnivores. *Anim. Cogn.* 24, 555-568. 10.1007/s10071-020-01438-4 33231749

[JEB243726C86] Stöwe, M., Bugnyar, T., Heinrich, B. and Kotrschal, K. (2006). Effects of group size on approach to novel objects in ravens (*Corvus corax*). *Ethology* 112, 1079-1088. 10.1111/j.1439-0310.2006.01273.x

[JEB243726C87] Thornton, A. and Lukas, D. (2012). Individual variation in cognitive performance: developmental and evolutionary perspectives. *Philos. Trans. R. Soc. B Biol. Sci.* 367, 2773-2783. 10.1098/rstb.2012.0214 PMC3427550 22927576

[JEB243726C88] Timm, R., Cuarón, A. D., Reid, F. and Helgen, K. (2008). Procyon lotor. In IUCN Red List of Threatened Species 2008. e.T41686A10512370. 10.2305/IUCN.UK.2016-1.RLTS.T41686A45216638.en.

[JEB243726C89] US Census Bureau (2021). QuickFacts Laramie city, Wyoming. Retrieved from https://www.census.gov/quickfacts/laramiecitywyoming

[JEB243726C90] Webster, M. M. and Rutz, C. (2020). How STRANGE are your study animals? *Nature* 582, 337-340. 10.1038/d41586-020-01751-5 32541916

[JEB243726C91] Wehtje, M. and Gompper, M. E. (2011). Effects of an experimentally clumped food resource on raccoon *Procyon lotor* home-range use. *Wildlife Biol.* 17, 25-32. 10.2981/10-012

[JEB243726C92] Western Regional Climate Center (2016). Laramie, Wyoming. Retrieved from https://wrcc.dri.edu/cgi-bin/cliMAIN.pl?wy5435

[JEB243726C93] Wright, T. F., Eberhard, J. R., Hobson, E. A., Avery, M. L. and Russello, M. A. (2010). Behavioral flexibility and species invasions: the adaptive flexibility hypothesis. *Ethol. Ecol. Evol.* 22, 393-404. 10.1080/03949370.2010.505580

[JEB243726C94] Zeveloff, S. I. (2002). *Raccoons: A Natural History*. UBC Press.

