# Supplementary material for: Environmental, individual and social traits of free-ranging raccoons influence performance in cognitive testing
Source: J Exp Biol. 2022 Sep 22;225(18):jeb243726. doi: 10.1242/jeb.243726 (PMC9637273; doi:10.1242/jeb.243726)
Supplement: Supplementary information [file jexbio-225-243726-s1.pdf]

Supplementary Materials and Methods. Additional methods and results.

Animal trapping and detection

We set up camera traps in locations where we expected raccoons to be present (e.g. proximity to water and vegetation) or where Laramie residents had reported seeing raccoons in the past. This included city parks and public spaces, as well as private property (e.g. residential backyards, local businesses) that we gained permission to access. We subsequently trapped sites with confirmed raccoon presence using Tomahawk live traps (81.28×25.4×25.4 cm, length×width×height) baited with wet cat food. Our general goal was to trap all resident raccoons in Laramie, including juveniles born each year, however priority was given to trapping sites on the West side of Laramie where we believe the highest density of raccoons to be and therefore planned to conduct our cognitive experiments.

Trapping and testing was conducted annually between the months of May and October. In 2018, trapping occurred before testing began, whereas in 2019 testing occurred before and after trapping. In addition, because we did not have access to one of our study sites in 2018, it had not been trapped since 2017. The time between trapping and testing, therefore, varied among individuals tested at different sites in different years. Specifically, some individuals first encountered the testing device shortly after trapping ended (min.=6 days), whereas others experienced a much longer duration between trapping and testing (max.=655). We suspected that active trapping efforts could have made raccoons in the area to be more risk-adverse, and therefore variation in the time between trapping and testing could have influenced whether raccoons habituated to the device or not. When plotted, however, no clear pattern was found between habituation (0, 1) and the time since trapping (days). Thus, we are confident that our trapping protocol did not affect habituation to the testing device.

Note regarding striped skunks

Skunks were opportunistically trapped and marked under the same permits used for our research on raccoons (University of Wyoming Institutional Animal Care and Use Committee: no. 20180813SB00321-02; Wyoming Game and Fish Department Chapter 33 Permit ID: 1019). Thus, free-ranging, PIT-tagged skunks within our study sites (n=14) could be detected by the testing device and participate in testing. We detected a total for four skunks during the experiment, one of whom learned to operate the testing device and completed serial reversals. Although reversal learning data from skunks is not included in the present study, our observations of free-ranging skunks during field testing, paired with our testing of skunks in captive conditions (Stanton et al., 2021), suggests that the methodology outlined here can be used with multiple, mesocarnivore species in both natural and captive testing conditions.

Journal of Experimental Biology • Supplementary information

Notable differences in captive versus natural conditions

Wild raccoons tested in captivity were all classified as adults at the time of trapping. They were tested once a night while alone in their home enclosure, and testing sessions lasted for 30 to 90 min. Individuals varied in their prior experience with the cognitive testing device, which likely influenced the number of errors being made initially but not across time (discussed in Stanton et al., 2021). Free-ranging raccoons, which constituted a mix of adults and juveniles, were able to start and stop testing at any point during a testing night, and they may have been subject to interruptions and/or social interference during testing. For these reasons we identified reversal events that were split between testing nights for free-ranging raccoons (e.g. started a reversal during one testing night but completed the reversal on a subsequent testing night) so that we could identify the effect of prolonged gaps in time (i.e. breaks) on performance. We also did not include data from natural conditions where high social interference may have affected the selections being made within the device. Similarly, if an individual experienced high social interference in the majority of their testing nights they were not included in the dataset (n=7). Lastly, captive testing was delivered in blocks of 10 selections, such that an individual was required to achieve nine out of 10 selections correct within a block. If not, then a new block of 10 selections was initiated. However, selections in wild conditions were not grouped into blocks and we instead used a sliding window format, in which the raccoon was required to make nine correct selections within 10 consecutive selections.

Table S1. Standardized points during the four phases of trapping when observations of raccoon behavior (i.e. vocalizations, movement, contact with humans) were made

Phase and observation points
**Phase 1 – Capture**
• Behavior when human first sees animal in trap
• Behavior when human first stands next to trap at capture site
• Behavior when human lifts trap cover and flags trap at capture site
• Behavior when human moves trap from capture site to truck
• Behavior when human moves trap from truck to injection site
• Behavior when human removes trap cover
**Phase 2 – Immobilization**
• Behavior when human approaches trap for injection
• Behavior when human probes raccoon with capped needle prior to injection
• Behavior when human injects raccoon with needle
**Phase 3 – Feeding**
• Behavior when human first sees animal in trap after recovery
• Behavior when human approaches trap and inserts cage divider
• Behavior to human opening trap and placing food and water in cage, shut door, and remove cage divider
**Phase 4 – Release**
• Behavior when human first sees animal in trap
• Behavior when human first stands next to trap
• Behavior when human moves trap from recovery site to truck
• Behavior when human moves trap from truck to release site
• Behavior when human puts trap on ground prior to opening door
• Behavior when human opens trap door at release site

Journal of Experimental Biology • Supplementary information

Table S2. Model summary for the effects of age and sex on several biological and behavioral traits

	Type	*N*	R^2	*P*-value	lower CI	upper CI	Beta	*t*-value
Model – Body condition score								
BCS ~ Age*Sex	GLM (Gaussian)	204	0.13					
Age-juvenile				1.90e-06	−0.133497969	−0.05730414	−0.09540	−4.908
Sex-male				0.0101	−0.100235853	−0.01403327	−0.05713	−2.598
Age-juvenile:Sex-male				0.0316	0.005316034	0.10703337	0.05617	2.165
Model – Trappability								
Trappability score ~ Age*Sex	GLM (quasibinomial)	182	0.03					
Age-juvenile				0.347	−0.4993908	0.1837688	−0.1640	−0.943
Sex-male				0.391	−0.1993357	0.5252940	0.1587	0.860
Age-juvenile:Sex-male				0.615	−0.3280522	0.5481791	0.1125	0.504
Model – Docility								
Vocalization ~ Age*Sex	CLM	178	NA					
Age-juvenile	(ordered)			0.4628	−0.5166814	1.153186	0.3117	0.734
Sex-male				0.1022	−0.1476393	1.687459	0.7627	1.634
Age-juvenile:Sex-male				0.0809	−2.1263523	0.117982	−0.9977	−1.746
Hosmer-Lemeshow goodness-of-fit test: P=0.36								
Model – Sociality								
Alone ~ Age*Sex	GLM (binomial)	40	0.17					
Age-juvenile				0.0258	−3.9005200	−0.3305036	−1.9924	−2.229
Sex-male				0.4254	−3.1526623	1.4290430	−0.8938	−0.797
Age-juvenile:Sex-male				0.5405	−2.0496705	3.7828333	0.8938	0.612

Journal of Experimental Biology • Supplementary information

Table S3. Repeatability across trapping phases and years

Across trapping phases (i.e. within a trapping event):	*P*-value	lower CI	upper CI	Beta	*z*-value
Model (Cumulative Link Mixed Model fitted with the Laplace approximation)					
Vocalization Score ~ Testing Phase + (1|Animal ID)					
Phase2_Immobilization	2e-16	1.966033	3.164286	2.5652	8.392
Phase3_Feeding	5.71e-10	1.270956	2.446464	1.8587	6.198
Phase4_Release	2.80e-08	1.046816	2.188757	1.6178	5.553
ICC=Intercept^2 / (Intercept^2 + Residual^2): 3.733853 ^2/(3.733853 ^2+3.289868^2)=0.5629606					
Movement Score ~ Testing Phase + (1|Animal ID)					
Phase2_Immobilization	0.000793	−1.2683350	−0.33291619	−0.8006	−3.355
Phase3_Feeding	0.066440	−0.8684571	0.02849198	−0.4200	−1.835
Phase4_Release	1.24e-08	0.8571170	1.75698505	1.3071	5.694
ICC=Intercept^2 / (Intercept^2 + Residual^2): 0.7397941 ^2/(0.7397941^2+3.289868^2)=0.0481328					
**Across trapping years (i.e. across trapping events):**	*P*-value	lower CI	upper CI	Beta	*z*-value
Model (Cumulative Link Mixed Model fitted with the Laplace approximation)					
Vocalization Score ~ Trapping year + (1|Animal ID)					
Trapping2	0.489	−0.8754376	1.8313787	0.4780	0.692
ICC=Intercept^2 / (Intercept^2 + Residual^2): 15.68937^2/(15.68937^2+3.289868^2)=0.9578829					
Movement Score ~ Trapping year + (1|Animal ID)					
Trapping2	0.879	−1.2323855	1.0548095	−0.08879	−0.152
ICC=Intercept^2 / (Intercept^2 + Residual^2): 0.4322022^2/(0.4322022^2+3.289868^2)=0.01696623					
Contact Score ~ Trapping year + (1|Animal ID)					
Trapping2	0.51	−1.7733538	3.569532	0.8981	0.659
ICC=Intercept^2 / (Intercept^2 + Residual^2): 1.800755^2/(1.800755^2+3.289868^2)=0.2305368					
	P-value	lower CI	upper CI	Beta	*t*-value
Model (Linear mixed model fit by REML)					
Trappability score ~ Trapping year + (1|Animal ID)					
Trapping2	0.000599	−0.05237997	−0.01660210	−0.034491	−3.836
ICC=Intercept^2 / (Intercept^2 + Residual^2): 0.0002763574 ^2/(0.0002763574 ^2+0.001253317^2)=0.04636625					
Trappability score ~ Trapping year + Age*Sex + (1|Animal ID)					
Trapping2	0.00139	−0.05341176	−0.01491103	−0.034275	−3.429
Trapping3	0.00198	−0.09272151	−0.02331963	−0.058523	−3.246
Age-juvenile	0.82481	−0.02764886	0.02225475	−0.002856	−0.222
Sex-male	0.71031	−0.02095081	0.03007033	0.004928	0.374
Age-juvenile:Sex-male	0.64057	−0.03364403	0.05249216	0.010242	0.469

Table S4. Results of individual models from natural conditions and captive conditions (reproduced from Stanton et al., 2021). Note that cells lacking data indicate models that could not be calculated due to data limitations.

AID	Species	Model (Poisson GLM)	*P*-value	lower CI	upper CI	Beta	d.f.	*z*-value	R^2
Natural Condition									
Allspice	Raccoon	Error ~ Reversal Number	0.000313	−0.1002505	−0.02979731	−0.06469	14	−3.604	0.14
Allspice	Raccoon	Error ~ Reversal Number + Side	0.012448	−0.74827235	−0.09326059	−0.41669	13	−2.499	0.20
Caper	Raccoon	Error ~ Reversal Number	0.90	−0.175318	0.1539761	−0.01050	4	−0.125	0.005
Caper	Raccoon	Error ~ Reversal Number + Side	0.92	−0.6532777	0.5501225	−0.03077	4	−0.101	0.004
Chia	Raccoon	Error ~ Reversal Number	0.000259	0.2503498	0.26641278	−0.03135	25	−3.653	0.22
Chia	Raccoon	Error ~ Reversal Number + Side	0.953099	−0.2503498	0.26641278	0.007743	24	0.059	0.22
Chive	Raccoon	Error ~ Reversal Number	0.119	−0.07121936	0.007951245	−0.03140	15	−1.558	0.05
Chive	Raccoon	Error ~ Reversal Number + Side	0.573	−0.27407842	0.50241236	0.11127	14	0.564	0.05
Coco	Raccoon	Error ~ Reversal Number	0.0248	−0.0416705	−0.002889005	−0.022172	26	−2.244	0.07
Coco	Raccoon	Error ~ Reversal Number + Side	0.9322	−0.32435160	0.297415588	−0.013461	25	−0.085	0.07
Okefenoke	Raccoon	Error ~ Reversal Number	0.0681	−0.03761509	0.00129984	−0.018097	21	−1.824	0.03
Okefenoke	Raccoon	Error ~ Reversal Number + Side	0.1368	−0.06126938	0.45832968	0.196937	20	1.488	0.05
Primrose	Raccoon	Error ~ Reversal Number	0.586	−0.07725152	0.1375734	0.02979	6	0.545	0.02
Primrose	Raccoon	Error ~ Reversal Number + Side	0.021	0.12797689	1.294357	0.68135	5	2.307	0.4
Tarragon	Raccoon	Error ~ Reversal Number	0.684	−0.07447412	0.1138773	0.01951	6	0.407	0.006
Tarragon	Raccoon	Error ~ Reversal Number + Side	0.000319	−1.63090947	−0.4988743	−1.03183	5	−3.600	.59
Tombigbee	Raccoon	Error ~ Reversal Number	0.359	−0.03609974	0.1003113	0.03186	8	0.917	0.007
Tombigbee	Raccoon	Error ~ Reversal Number + Side	3.63e-11	−4.08943445	−2.2470621	−3.04527	7	−6.619	0.90
Voyageurs	Raccoon	Error ~ Reversal Number	0.196	−0.8969634	0.1698147	−0.3477	1	−1.293	0.64
Voyageurs	Raccoon	Error ~ Reversal Number + Side	-	-	-	-	-	-	-
Captive Condition									
Vega	Raccoon	Error ~ Reversal Number	3.76E-05	−0.05833	−0.02084	−0.03936	30	−4.12153	0.320028
Vega	Raccoon	Error ~ Reversal Number + Side	0.097243	−0.05013	0.622547	0.283916	29	0.097243	0.370285
Pollux	Raccoon	Error ~ Reversal Number	3.24E-06	−0.05743	−0.02348	−0.04027	30	−4.65468	0.324268

Journal of Experimental Biology • Supplementary information

AID	Species	Model (Poisson GLM)	*P*-value	lower CI	upper CI	Beta	d.f.	*z*-value	R^2
Pollux	Raccoon	Error ~ Reversal Number + Side	0.383205	−0.43662	0.1698	−0.13465	29	0.383205	0.33515
Rigel	Raccoon	Error ~ Reversal Number	7.52E-05	−0.11835	−0.04034	−0.0786	18	−3.95933	0.363819
Rigel	Raccoon	Error ~ Reversal Number + Side	0.004783	0.193569	1.059276	0.620657	17	0.004783	0.541853
Sirius	Raccoon	Error ~ Reversal Number	0.00045	−0.07276	−0.02075	−0.04648	21	−3.50888	0.309757
Sirius	Raccoon	Error ~ Reversal Number + Side	0.183774	−0.10804	0.583329	0.233878	20	0.183774	0.353527
Oberon	Raccoon	Error ~ Reversal Number	1.15E-05	−0.04929	−0.0189	−0.034	26	−4.38788	0.261329
Oberon	Raccoon	Error ~ Reversal Number + Side	0.586995	−0.17394	0.310216	0.067008	25	0.586995	0.265255
Astrid	Raccoon	Error ~ Reversal Number	0.006162	−0.10381	−0.01741	−0.06029	11	−2.73905	0.221065
Astrid	Raccoon	Error ~ Reversal Number + Side	0.250413	−0.13142	0.517668	0.189896	10	0.250413	0.259792
Castor	Raccoon	Error ~ Reversal Number	7.43E-15	−0.08426	−0.05042	−0.06707	29	−7.77688	0.392246
Castor	Raccoon	Error ~ Reversal Number + Side	0.001128	0.18844	0.751212	0.466755	28	0.001128	0.456132
Luna	Raccoon	Error ~ Reversal Number	-	-	-	-	-	-	-
Luna	Raccoon	Error ~ Reversal Number + Side	-	-	-	-	-	-	-

Table S5. Summary table for captive versus natural testing conditions

Model (Poisson GLMM w/ log link)	*P*-value	lower CI	upper CI	Beta	*z*-value
All individuals					
Errors~Reversal*Condition+(1 + Test | Animal ID)					
R^2=0.67, *d. f.*=322					
(Intercept)	< 2e-16	2.206773424	2.60344750	2.410069	25.184
Reversal	< 2e-16	−0.060474603	−0.03791021	−0.048339	−9.307
ConditionNatural	0.46246	−0.362887765	0.17420840	−0.095314	−0.735
Reversal:ConditionNatural	0.00437	0.006659671	0.04111761	0.022674	2.850
Adults only					
Errors~Reversal*Condition+(1 + Test | Animal ID)					
R^2=0.71, *d.f.*=231					
(Intercept)	<2e-16	2.190584725	2.61736705	2.411200	24.202
Test	<2e-16	−0.062001121	−0.03782622	−0.048766	−8.960
ConditionNatural	0.1740	−0.676581780	0.15644441	−0.266346	−1.359
Test:ConditionNatural	0.0195	0.004615396	0.05764457	0.028737	2.336

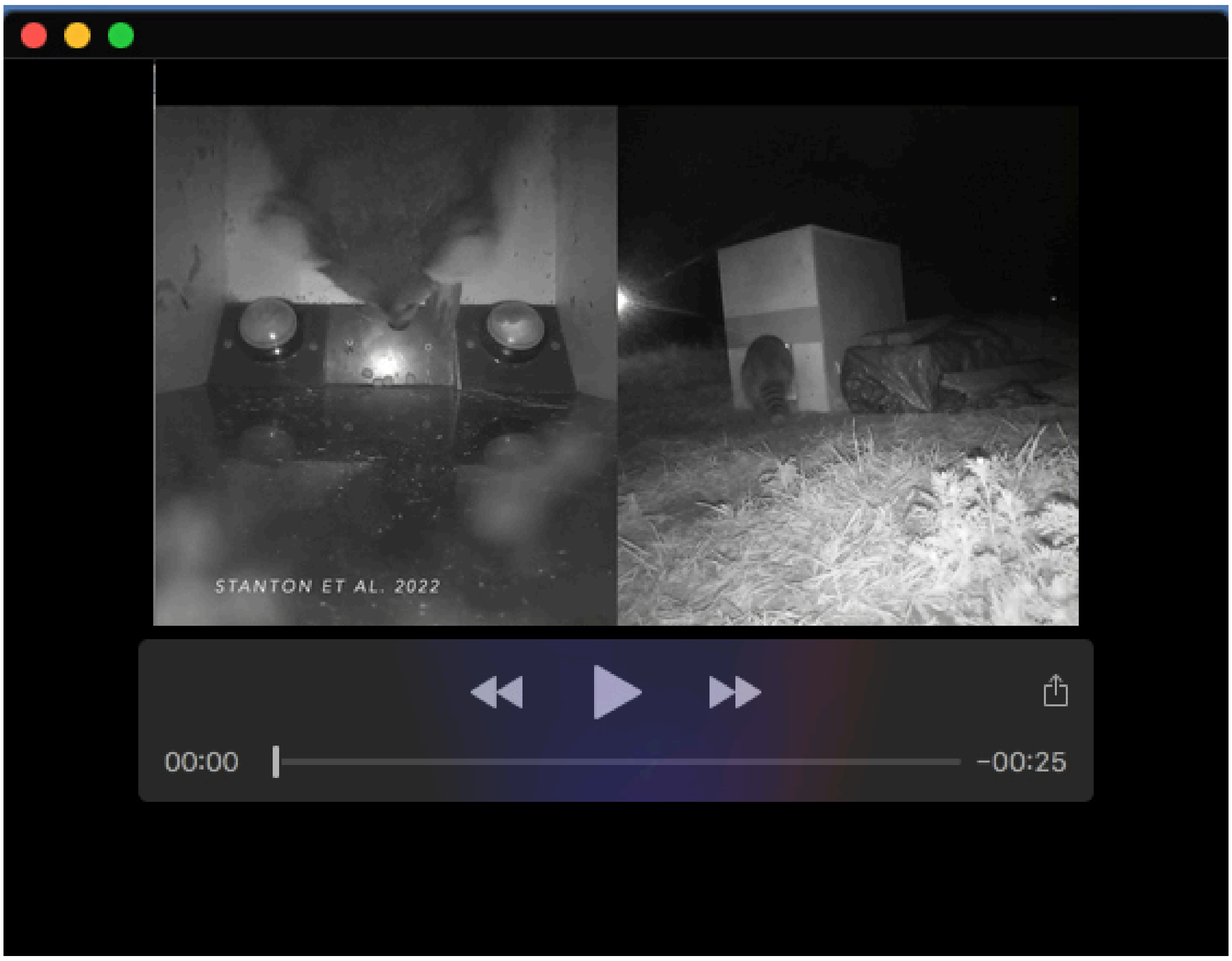

Movie 1. Video footage from a testing session. As seen in the video, a raccoon is eating dog kibble (i.e., the food reward) inside of the testing device and then moves outside of the device to investigate his surroundings. He then moves back inside of the device and pushes on the button to his left, which is the incorrect choice. In response, a low-pitched sound is played as the lights in both LED buttons shut off and the device becomes unresponsive for two seconds. After the two second time-out period has passed, the raccoon makes the correct selection by pushing on the button to his right side and a small amount of dog kibble is released.

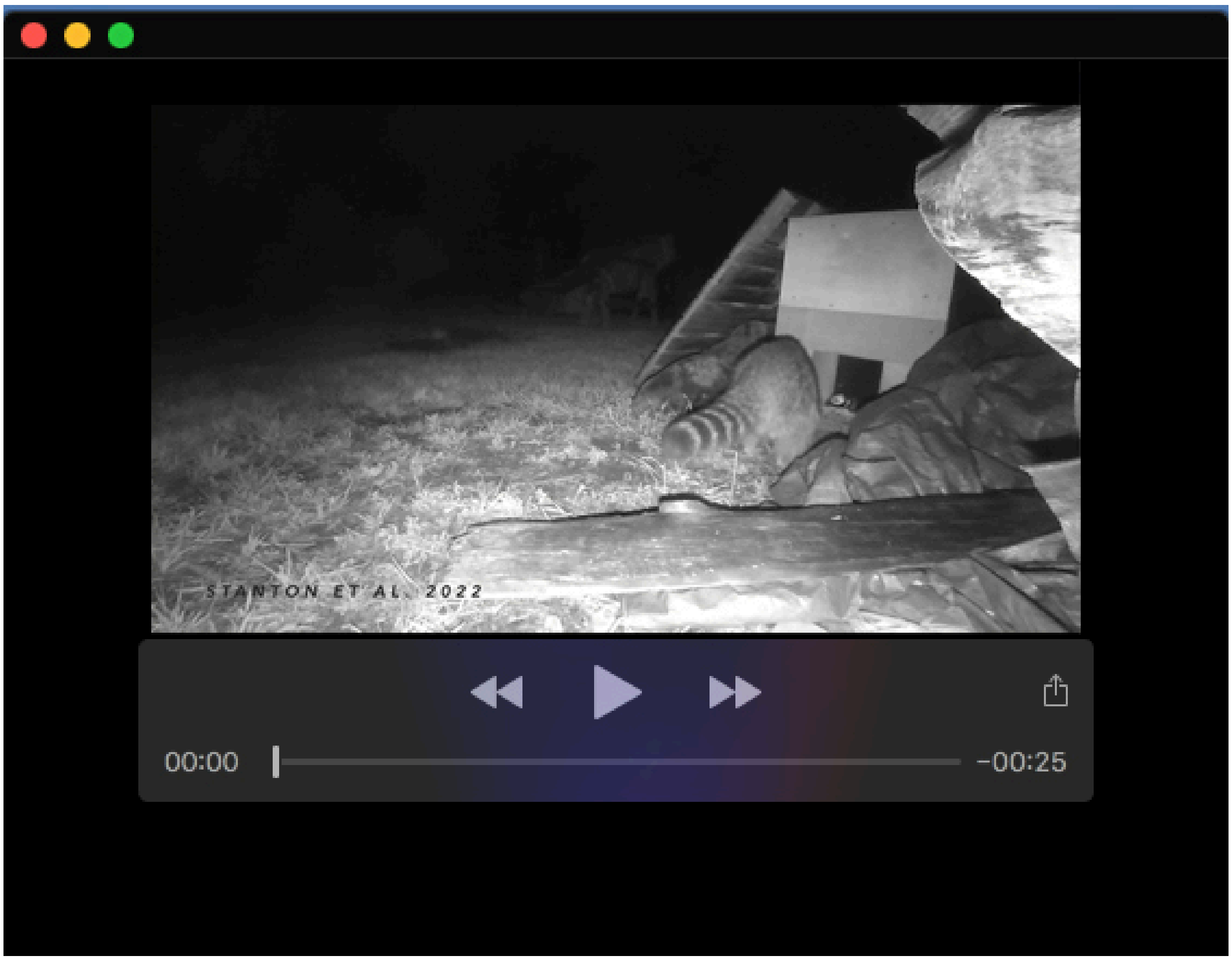

Movie 2. Video footage illustrating the process of habituation to the testing device. As the raccoon approaches the testing device, her PIT tag is detected, and the device automatically releases a food reward. The raccoon reacts by swiftly moving away from the device, but after a moment she returns to collect the food reward. Upon detection of her PIT tag again, the device releases a second food reward. This time the raccoon reacts by flinching at the sound of the device activating, but does not move away from the device as she did before. Finally, the device releases a third reward and although the raccoon flinches again, it appears less than her previous reaction.

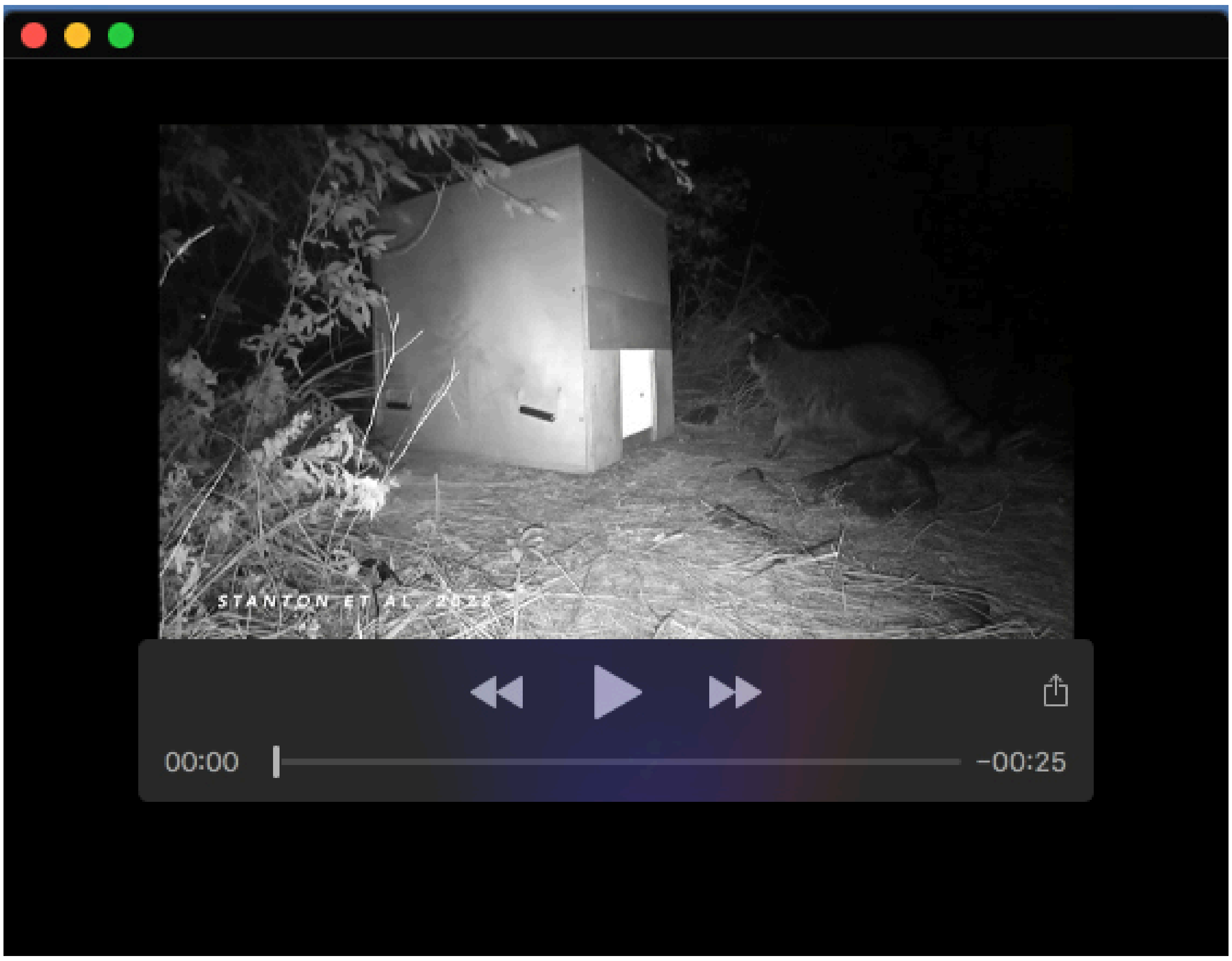

Movie 3. An example of a raccoon that never habituates to the testing device. The raccoon is seen approaching the device for the first (and only) time. When his PIT tag is detected, the device activates and releases a food reward. In response, the raccoon runs away and is not detected again by a testing device for the remainder of the study.ud
